# *BCL2A1* marks an early, less-exhausted CD8^+^ tumor-infiltrating lymphocyte state associated with PD-1 blockade response in NSCLC

**DOI:** 10.3389/fimmu.2026.1908216

**Published:** 2026-10-07

**Authors:** Hoang Minh Quan Pham, Chia-Ling Chen, Po-Hao Feng, Kang-Yun Lee, Yu-Ping Hung, Chiou-Feng Lin

**Affiliations:** 1 International Ph.D. Program in Medicine, College of Medicine, Taipei Medical University, Taipei, Taiwan; 2 Department of Oncology, Faculty of Medicine, Can Tho University of Medicine and Pharmacy, Can Tho, Vietnam; 3 Department of Microbiology and Immunology, School of Medicine, College of Medicine, Taipei Medical University, Taipei, Taiwan; 4 School of Respiratory Therapy, College of Medicine, Taipei Medical University, Taipei, Taiwan; 5 Division of Pulmonary Medicine, Department of Internal Medicine, Shuang Ho Hospital, Taipei Medical University, New Taipei, Taiwan; 6 Division of Pulmonary Medicine, Department of Internal Medicine, School of Medicine, College of Medicine, Taipei Medical University, Taipei, Taiwan; 7 Graduate Institute of Clinical Medicine, College of Medicine, Taipei Medical University, Taipei, Taiwan; 8 TMU Research Center of Thoracic Medicine, Taipei Medical University, Taipei, Taiwan; 9 Graduate Institute of Medical Sciences, College of Medicine, Taipei Medical University, Taipei, Taiwan; 10 Core Laboratory of Immune Monitoring, Office of Research & Development, Taipei Medical University, Taipei, Taiwan

**Keywords:** BCL2A1, CD8+ TIL, immune checkpoint blockade, NSCLC, T-cell exhaustion

## Abstract

**Background:**

The tumor microenvironment exerts intense survival pressure on CD8^+^ tumor-infiltrating lymphocytes (TILs), yet the cellular basis for an earlier, less-exhausted state associated with response to immune checkpoint blockade (ICB) in non-small-cell lung cancer (NSCLC) remains unclear. We previously identified *BCL2A1* as a CD8^+^ TIL-derived marker predictive of a response to PD-1 blockade. Here we combine deep single-cell discovery with experimental validation in primary human T cells to characterise the BCL2A1-associated CD8^+^ TIL state and its candidate upstream signal.

**Methods:**

We integrated four transcriptomic cohorts—pre-treatment bulk RNA-seq with CD8^+^ TIL deconvolution (n=60), on-treatment scRNA-seq/TCR-seq (n=11), longitudinal scRNA-seq/TCR-seq (n=18), and an independent post-treatment cohort (n=59)—with in silico perturbation, pathway, and metabolic-flux analyses, and experimentally tested a key prediction of this state by flow cytometry in primary human CD8^+^ T cells.

**Results:**

*BCL2A1*^high^ CD8^+^ TILs showed elevated BCL2A1 expression without coordinated upregulation of anti-apoptotic BCL2-family genes, maintained minimal exhaustion, and occupied an early, *BCL6*-, *TCF7*-, and *NFKB1*-regulated pseudotime state. Across longitudinal and post-treatment cohorts, they formed diverse, low-expansion repertoires rather than dominant expanded clones, with reduced biosynthetic flux (branched-chain amino acid and purine metabolism) consistent with a non-proliferative state. NF-κB signaling was elevated, and TNF-α was nominated as the leading candidate upstream ligand of *BCL2A1*. We then experimentally tested this prediction: in primary human CD8^+^ T cells, TNF-α induced *BCL2A1* protein ≈13-fold (44.5% vs. 3.4% BCL2A1^+^; MFI 2.93-fold; P < 0.0001), whereas CD3/CD28 activation alone did not, establishing a TNF-α-specific, protein-level response.

**Conclusions:**

Combining integrative single-cell analysis with experimental validation in primary human T cells, we define *BCL2A1* as a marker of an early, less-exhausted CD8^+^ TIL state associated with improved ICB outcomes in NSCLC; a survival-related role is supported by these associations but was not functionally tested.

## Introduction

Immune checkpoint blockade (ICB) therapy has revolutionized the treatment landscape of non-small cell lung cancer (NSCLC), offering durable clinical benefit in a subset of patients (1, 2). However, primary and acquired resistance remain pervasive challenges, often linked to the functional collapse of cytotoxic CD8^+^ tumor-infiltrating lymphocytes (TILs)—the principal effectors of antitumor immunity (3, 4). Persistent antigenic stimulation within the tumor microenvironment drives progressive dysfunction of CD8^+^ T cells, culminating in an “exhausted” state characterized by diminished proliferative capacity, impaired cytokine secretion, and sustained expression of inhibitory receptors such as PD-1, TIM-3, and LAG-3 (5, 6). While exhaustion has been extensively characterized as a terminal dysfunctional state, recent studies have revealed a continuum of transcriptional and functional states among TILs, including transitional or resilient subsets that retain effector potential under chronic stimulation (7, 8).

Among these resilient subsets, mechanisms that protect T cells from activation-induced cell death (AICD) or apoptosis may critically shape the persistence and effectiveness of antitumor responses (9, 10). Anti-apoptotic programs not only prolong T-cell survival but also preserve the effector reservoir necessary for sustained immune control, particularly under therapeutic pressure from ICB. In this context, *BCL2A1*, a pro-survival member of the *BCL2* family and a transcriptional target of NF-κB, plays a pivotal role. Unlike canonical *BCL2* or *MCL1*, *BCL2A1* expression is transient, inducible under inflammatory conditions, and specifically implicated in promoting lymphocyte survival during robust immune activation (11–14). Therefore, we posit that *BCL2A1* may serve as a critical survival switch, enabling effector T cells to withstand the harsh inflammatory milieu of the tumor microenvironment.

Building on this rationale, our previous work identified elevated *BCL2A1* expression specifically within CD8^+^ TILs as an immune-intrinsic biomarker predictive of PD-1 blockade responsiveness in NSCLC (15). This finding suggests that BCL2A1 marks a subset of T cells that remains distant from terminal exhaustion. We hypothesize that these *BCL2A1*-high CD8^+^ TILs represent a distinct, less-exhausted state that preserves immune competence under prolonged stimulation, thereby contributing to durable ICB responses. To test this hypothesis and elucidate the mechanisms driving this BCL2A1-associated phenotype, we integrated multiple complementary transcriptomic datasets encompassing diverse clinical and biological contexts: (i) a bulk RNA-seq cohort of pre-treatment NSCLC tumors with defined ICB outcomes; (ii) an on-treatment single-cell RNA/TCR dataset to delineate real-time transcriptional phenotypes of CD8^+^ TILs under PD-1 blockade; (iii) a longitudinal single-cell RNA/TCR dataset to trace clonal dynamics and recurrence across pre- and post-treatment samples; and (iv) an independent post-treatment single-cell RNA/TCR dataset to independently evaluate the transcriptional and clonal characteristics of BCL2A1-high TILs after prolonged therapy. Finally, we experimentally validated a key prediction of this analysis in primary human CD8^+^ T cells.

## Materials and methods

### Data and code availability

We performed an integrated analysis across four transcriptomic datasets. The raw data and processed count matrices are available on the Gene Expression Omnibus (GEO) under accession codes GSE161537, GSE176021, GSE179994, and GSE243013. These included: bulk RNA-seq from GSE161537 (pre-treatment LUAD samples, n = 60), single-cell RNA-seq from GSE176021 (on-treatment, n = 11), a longitudinal scRNA-seq and TCR-seq cohort from GSE179994 (pre- and post-treatment, n = 18), and an independent post-treatment scRNA-seq and TCR-seq cohort from GSE243013 (n = 59) (**Supplementary Table S1**).

### Definition of *BCL2A1*^high^ cells

Given the ~10% dropout rate of *BCL2A1*, we focused on high-expression quantiles (≥ 90th percentile) to define *BCL2A1*^high^ cells. Across these thresholds (gmm_high, 95th, 97th, 99th), the same core Hallmark programs were consistently enriched, indicating that a single arbitrary cut-off does not drive our conclusions. Notably, this restricted definition identifies a rare subpopulation (~5-10% of CD8^+^ T cells) that remains biologically coherent across independent datasets. For consistency and interpretability, all single-cell figures presented in the main text use the 95th percentile (q95) as the operational cut-off to define *BCL2A1*^high^ cells. Because *BCL2A1* is itself a member of the anti-apoptotic gene set used for scoring, we performed a leave-one-out ablation (re-scoring with *BCL2A1* removed) to test whether the anti-apoptotic signal was *BCL2A1*-specific or program-wide.

### Bulk RNA-seq analysis (GSE161537)

Pre-treatment LUAD samples (n = 60) were analyzed to define baseline tumor immune composition. Expression values were obtained as log2-transformed counts per million (log2CPM) from the GEO-deposited panel matrix; raw counts were not available for this dataset. To quantify the tumor immune contexture, we applied a computational deconvolution approach to the bulk RNA-seq data. Specifically, the ‘BCL2A1_Immune’ score was calculated using the EPIC algorithm (Estimating the Proportions of Immune and Cancer cells). This score is a composition-informed estimate at the tissue level and is not a compartment-resolved measurement of *BCL2A1* expression; the cellular origin of the *BCL2A1* signal is addressed with the single-cell cohorts. Concurrent immune activation was assessed using the ‘Hot_score’, a composite transcriptomic signature derived from the mean log2(CPM + 1) expression of 27 immune-activation genes (including *CCL19*, *CCR2*, *CCR4*, *CCR5*, *CD27*, *CD40LG*, *CD8A*, *CXCL10*, *CXCL11*, *CXCL13*, *CXCL9*, *CXCR3*, *CXCR6*, *FASLG*, *FGL2*, *GZMA*, *GZMH*, *IDO1*, *IFNG*, *IRF8*, *LAG3*, *LYZ*, *MS4A1*, *PDCD1*, *TBX21*, *TLR7*, and *TLR8*) previously established by Foy et al. to define immune-hot tumor microenvironments. Pathway enrichment analysis was performed using single-sample gene set enrichment analysis (ssGSEA). To test whether the elevated cytotoxic and exhaustion scores in *BCL2A1*^high^ tumors merely reflected higher CD8 infiltration, we adjusted for the EPIC-estimated CD8 fraction using three independent strategies — covariate inclusion, residualization, and proportional down-weighting by (1 − CD8 fraction) in linear space — on the samples with a non-zero CD8 estimate.

Five T-cell state signatures were scored by ssGSEA: CD8 T-cell exhaustion and terminal exhaustion from MSigDB C7, and marker panels for progenitor exhaustion, NK-like CD8 and proliferative exhausted CD8. Because this dataset is a targeted panel of 2,559 genes, scoring used the signature members represented on it (48 of 200, 44 of 200, 5 of 6, 3 of 8 and 6 of 6 respectively).

### Single-cell RNA-seq analysis

#### Preprocessing

Raw 10x matrices were read and concatenated across samples in Scanpy. Cells with 200 to 8,000 detected genes and no more than 20% mitochondrial counts were retained, genes detected in fewer than three cells were removed, and doublets were excluded using Scrublet (expected doublet rate 0.06). Counts were normalized to 10,000 per cell and log1p-transformed. CD8^+^ T cells were defined as the intersection of CellTypist calls from the Immune_All_Low and Human_Lung_Atlas models (majority voting). Within this compartment, 2,000 highly variable genes (Seurat flavor) were scaled (maximum value 10), principal components were computed (arpack solver), and a neighbor graph (15 neighbors, 30 components) supported Leiden clustering (resolution 0.6) and UMAP embedding. No batch correction was applied. The same parameters were used for all three single-cell cohorts.

#### Discovery cohort GSE176021

The on-treatment dataset (n = 11) was processed to dissect transcriptional programs in individual CD8^+^ T cells during PD-1 blockade, yielding 137,435 CD8^+^ T cells for downstream analyses (16). AUCell scoring (17) was used to compute per-cell pathway enrichment. Pseudotime trajectories were constructed using Diffusion Pseudotime (DPT). The root was the first cell of Leiden cluster 0 in the object’s cell ordering; this cluster corresponds to a *GZMK*^+^ effector-memory population characterized by high memory-associated and low exhaustion-associated signature scores among the CD8^+^ T-cell clusters. Neither *BCL2A1* expression nor response group entered the selection. Regulatory networks were inferred using pySCENIC (18, 19).

#### Longitudinal validation cohort GSE179994

The longitudinal validation dataset GSE179994 (n = 18) included paired pre- and post-treatment LUAD samples with matched scRNA-seq and TCR-seq data (48,253 CD8^+^ T cells) (20). Preprocessing followed the same filtering criteria as GSE176021. AUCell pathway scores were compared between the *BCL2A1*^high^ and *BCL2A1*^low^ groups. Pseudotime distributions and enrichment in defined transcriptional states (Early Tex, Pre Tex, and ICB Tex; states defined from GSE176021 and applied to GSE179994) were quantified.

#### Independent validation cohort GSE243013

The independent post-treatment dataset GSE243013 (n = 59) was used to externally validate the BCL2A1-associated CD8^+^ TIL state (68,181 CD8^+^ T cells). Preprocessing and normalization followed the same pipeline. Pathway and gene-level comparisons focused on apoptosis, cytotoxicity, exhaustion, and IFN-γ programs. Subtype composition (Trm, Tem, Tm, and Tex) was assessed using cluster annotations from the source study. Regulon inference using pySCENIC was repeated to confirm the transcriptional architecture underlying *BCL2A1*^high^ cells. Pseudotime distributions and enrichment in defined transcriptional states (Early Tex, Pre Tex, and ICB Tex; states defined from GSE176021 and applied to GSE243013) were quantified.

### TCR repertoire analysis

TCR repertoire analysis was performed on all three single-cell cohorts with matched VDJ sequencing data. For the discovery cohort GSE176021, cells were matched to the processed CD8^+^ T cell dataset (n = 11; 68,566 matched cells) based on cell barcodes. For GSE179994 (n = 18; 25,665 matched cells), TCR repertoire analysis used unique TRA-TRB CDR3 pairs to assess clonotype expansion (defined at clone-size thresholds of ≥ 2 and ≥ 3 cells) and clonal persistence across pre- and post-treatment samples (20, 21). For GSE243013 (n = 59; 45,348 matched cells), TCR-seq data were used to calculate clonal diversity, expansion rates, and the fraction of clones with ≥ 2 and ≥ 3 cells.

TCR repertoire diversity was assessed using Shannon entropy, calculated as H = -Σ(p_i_ × log_2_(p_i_)), where p_i_ represents the frequency of each unique clonotype. Normalized Shannon entropy (Pielou’s evenness) was computed as H/H_max_. Clonality was defined as 1 minus the normalized entropy. Bootstrap resampling (n = 1,000 iterations) was performed to estimate 95% confidence intervals for entropy measurements. To test whether the raw Shannon entropy difference was attributable to unequal sampling depth, the *BCL2A1*^low^ compartment was subsampled without replacement within each patient to the corresponding *BCL2A1*^high^ cell count, Shannon entropy was recomputed on the subsample, and this was repeated 1,000 times (seed 42); per-patient medians were compared by paired Wilcoxon signed-rank test (n = 11). *BCL2A1*^high^ cells were defined using the 95th percentile threshold, consistent with the scRNA-seq analysis pipeline.

### *In silico* perturbation analysis using CellOracle

To assess the predicted consequences of perturbing transcription factors and *BCL2A1* on CD8^+^ T cell states, we performed *in silico* perturbation analysis using CellOracle (v0.18.0) on the discovery cohort (GSE176021) (22). Gene regulatory networks were constructed using a hybrid approach combining SCENIC-derived edges for eight canonical transcription factors (*RELA*, *RELB*, *NFKB1*, *TCF7*, *TOX*, *TBX21*, *EOMES*, *BCL6*) with correlation-based edges for *BCL2A1*. Perturbation simulations were performed for both knockout (KO) and overexpression (OE) conditions for all 9 regulators.

### Pathway activity analysis using PROGENy

Pathway activity scores were calculated using PROGENy (via decoupleR) with weighted pathway-gene matrices for 14 cancer-relevant signaling pathways: Androgen, EGFR, Estrogen, Hypoxia, JAK-STAT, MAPK, NFkB, PI3K, p53, TGFb, TNFA, Trail, VEGF, and WNT (23, 24). This analysis was performed on both the discovery cohort (GSE176021) and the longitudinal validation cohort (GSE179994). For GSE179994, pathway scores were calculated separately for pre-treatment (n = 22,057 cells) and post-treatment (n = 26,196 cells) samples to assess the temporal stability of pathway signatures. For the per-patient paired display, the available cells were pooled within each patient to give one value per patient per compartment (n = 18), and *BCL2A1*^high^ and *BCL2A1*^low^ values were compared by paired Wilcoxon signed-rank test with Benjamini–Hochberg correction across the 14 pathways.

### Upstream ligand and metabolic analysis

To identify upstream signals driving *BCL2A1* expression, we performed ligand-receptor-target network analysis using NicheNet (nichenetr v2) (25) on the discovery cohort (GSE176021), testing 121 candidate ligands relevant to T cell biology and NF-κB signaling.

Single-cell metabolic flux was estimated using scFEA with the Human-M168 metabolic module set (26) (168 reaction modules), which were aggregated into 22 metabolic super-modules for interpretation. Flux was estimated for GSE176021, GSE179994, and GSE243013. Per-module flux was averaged within each super-module and compared between *BCL2A1*^high^ and *BCL2A1*^low^ cells at the patient level (paired Wilcoxon signed-rank with Benjamini–Hochberg correction across super-modules), as detailed in the Statistical Analysis section.

### Cross-platform TNF-α pathway validation

To assess transcriptional evidence for the candidate TNF-α/NF-κB/BCL2A1 axis, two independent scRNA-seq datasets were analyzed. GSE205095 profiled 50,037 PBMCs from juvenile idiopathic arthritis with 24 h TNF-α stimulation (10 ng/mL) using 10X Chromium. GSE289019 profiled healthy-donor PBMCs from a rheumatoid arthritis study with 6 h TNF-α stimulation (5 ng/mL) using BD Rhapsody CITE-seq, yielding 18,085 cells for analysis. Both analyses were restricted to healthy donor samples. TNF-α-responsive cells were classified by composite NF-κB score (*NFKB1* + *RELA* + *RELB* > 0.1); CD8^+^ T cells were identified by detectable CD8A or CD8B expression (summed normalized expression > 0). In GSE289019 the CD8 criterion retained 10,682 of 18,085 cells, of which 9,219 also met the NF-κB criterion. Differential expression of five BCL2 family members (*BCL2A1*, *BCL2*, *MCL1*, *BCL2L1*, and *BCL2L2*) was evaluated by Wilcoxon rank-sum tests; Fisher’s exact tests assessed cell frequency changes.

### Flow cytometry validation of TNF-α-induced *BCL2A1* in primary CD8^+^ T cells

To validate the TNF-α→BCL2A1 axis at the protein level, peripheral blood mononuclear cells (PBMCs) from three healthy donors were isolated using SepMate™-50 tubes (STEMCELL Technologies, BC, Canada) following the manufacturer’s protocol. The study was approved by the Taipei Medical University Joint Institutional Review Board (TMU-JIRB No. N202504079), and all procedures involving human participants were conducted in accordance with the Declaration of Helsinki and institutional ethical guidelines. Isolated PBMCs were pre-activated with soluble anti-CD3/anti-CD28 antibodies in RPMI-1640 (10% FBS, 2 mM L-glutamine) for 24 h, then cultured for an additional 48 h under three conditions: vehicle (Resting), anti-CD3/CD28 alone (Activated), or anti-CD3/CD28 plus recombinant human TNF-α at 5 ng mL^-^¹ (n = 3 donors per condition). Cells were stained with Zombie R718 viability dye and surface markers (CD3, CD56, CD4, and CD8), fixed and permeabilized, and intracellular BCL2A1 was detected with a biotinylated anti-BCL2A1 antibody (streptavidin-APC). Data were acquired on an Attune NxT cytometer (Thermo Fisher Scientific). On CD8^+^ T cells gated as Lymphocytes → Live → CD3^+^CD56^-^ → CD4^-^CD8^+^, two endpoints were quantified: the percentage of BCL2A1^+^ events (fixed gate applied uniformly across samples) and BCL2A1 median fluorescence intensity (MFI). Groups were compared using a one-way ANOVA with Tukey’s HSD *post hoc* test.

### Statistical analysis

All statistical tests were two-sided, with P < 0.05 as the significance threshold, and the Benjamini–Hochberg procedure was used to control the false discovery rate in multiple-comparison settings. For single-cell pathway and gene-set analyses, primary comparisons between *BCL2A1*^high^ and *BCL2A*1^low^ groups were made at the patient level (pseudobulk) using paired Wilcoxon signed-rank tests, with Cohen’s d reported as the effect size; cell-level distributions are shown for visualization. Correlations were assessed using Pearson’s coefficient for the bulk immune-score analyses and Spearman’s ρ for monotonic pathway and ligand associations. TCR repertoire diversity was quantified by Shannon entropy and Pielou’s evenness, with 95% confidence intervals estimated by bootstrap resampling; differences in clonal expansion frequency were tested using Fisher’s exact test. Pathway activities (PROGENy via decoupleR) were tested with the corresponding linear-model statistics and corrected across the pathway family. For the cross-platform TNF-α analyses, differential expression was assessed using Wilcoxon rank-sum tests, and cell-frequency changes were assessed using Fisher’s exact test.

## Results

### Bulk RNA-seq (GSE161537) characterization of the *BCL2A1*^high^ immune phenotype in LUAD patients before ICB treatment

In our preliminary analysis of the discovery cohort, *BCL2A1*_Immune showed a statistically significant positive correlation with the Hot_score (r = 0.622, P = 1.1×10^-7^; **Figure 1A**), indicating an immune-hot phenotype in LUAD patients. In the *BCL2A1*^high^ group, ssGSEA revealed enrichment of cytotoxic, IFN-γ signaling, pyroptotic, exhaustion, and anti-apoptotic programs (Δ ssGSEA = +0.10 to +0.16), alongside reduced autophagy, all significant after FDR correction (q < 0.05; **Figure 1B**). Gene-level analysis revealed upregulation of *TNF* (q = 1.3×10^-4^), *FASLG* (q = 6.2×10^-4^), and *IL1B* (q = 1.3×10^-4^), indicating partial engagement of inflammatory caspase signaling (**Supplementary Table S2**). IFN-γ signaling was broadly elevated, with *IFNG* (q = 9.8×10^-4^), *STAT1* (q = 0.013), *CXCL9* (q = 2.9×10^-^³), and *CXCL10* (q = 8.5×10^-^³) all significantly increased in the *BCL2A1*^high^ group. Cytotoxicity-associated genes were likewise significantly higher — *GZMB* (q = 9.2×10^-4^), *PRF1* (q = 7.9×10^-5^), *GNLY* (q = 0.021), and *GZMA* (q = 3.2×10^-4^). Hierarchical pathway scoring (**Figure 1C**) revealed that T cell–mediated effector and cytotoxic programs were enriched both in the *BCL2A1*^high^ group (upper panel) and in ICB responders (lower panel), indicating partial concordance between *BCL2A1*-associated immune activation and responder-associated immune programs. IFN-γ pathway activity was significantly higher in ICB responders (P = 0.0417, Cohen’s d = 0.60; **Figure 1D**). Five exhaustion-related signatures (Tpex, Tex, P-Tex, Texterm, and NK-like CD8) were examined in ICB responders (**Figure 1E**) and in *BCL2A1*^high^ cells (**Figure 1F**). Pyroptosis scores correlated with *BCL2A1*, *GZMB*, and *PRF1* (ρ = 0.43–0.65), but not with apoptosis, autophagy, or ferroptosis (**Figure 1G**). These bulk cytotoxic and exhaustion signals were robust to adjustment for CD8 T-cell content across three independent strategies (5–11% attenuation; all P < 10^-^³; **Supplementary Table S3**), indicating they are not attributable solely to CD8 infiltration. Together, these features indicate that *BCL2A1*^high^ tumors display an inflamed, cytotoxic-leaning immune profile that resembles patterns observed in ICB responders.

**Figure 1:**
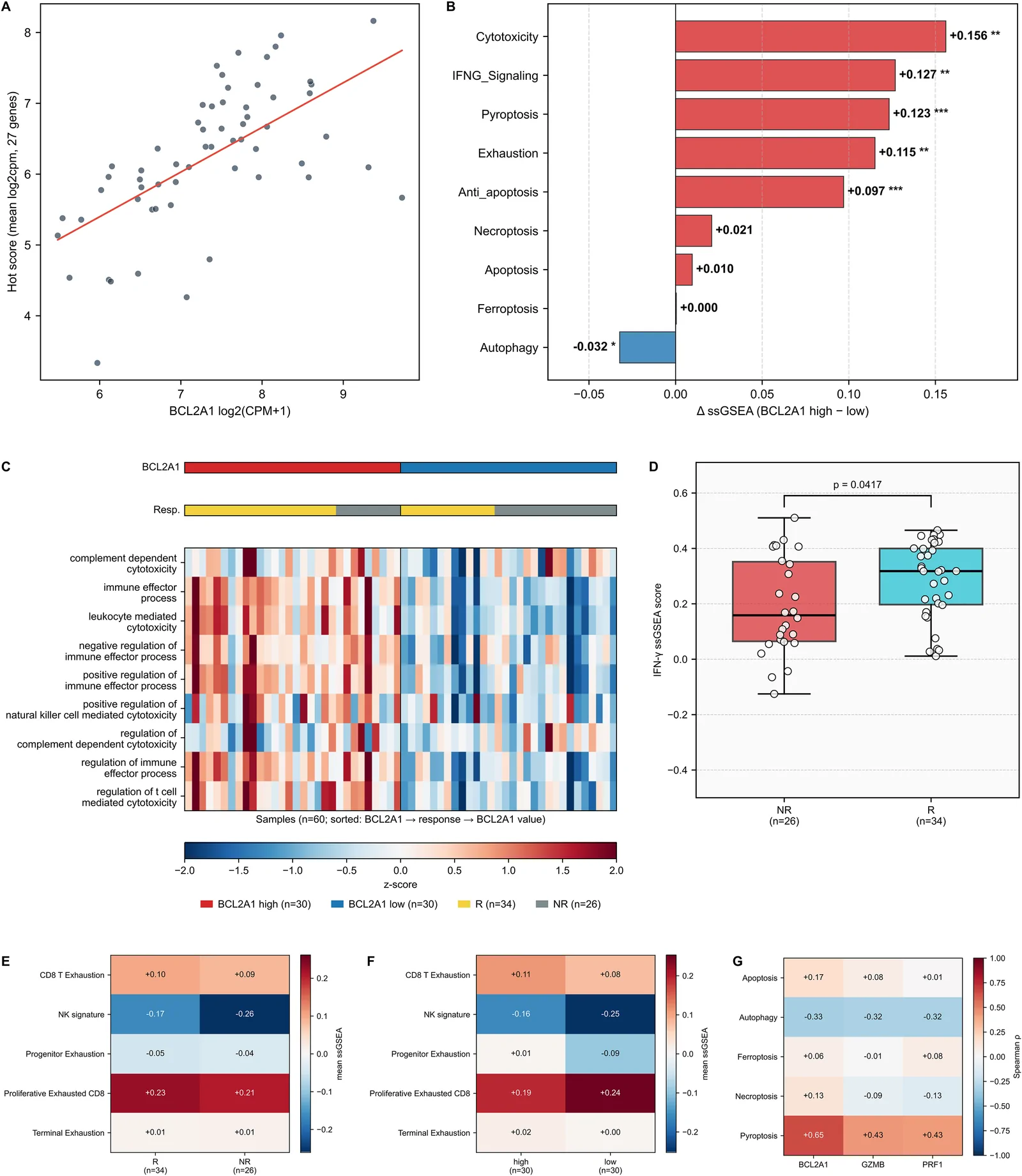
Pathway activity and gene expression profiles in CD8^+^ TILs across response and *BCL2A1* expression levels in the bulk cohort (GSE161537). **(A)** Correlation between *BCL2A1*_Immune and Hot_score. **(B)** ssGSEA enrichment differences (Δ, *BCL2A1* high − low) across nine cell-death and immune pathways; *q < 0.05, **q < 0.01, ***q < 0.001. **(C)** Hierarchical clustering of T cell–mediated effector and cytotoxicity gene-ontology programs, annotated by *BCL2A1* group and ICB response. **(D)** IFN-γ ssGSEA score in responders (R) versus non-responders (NR). **(E)** Five exhaustion-related signatures in responders versus non-responders. **(F)** The same five exhaustion-related signatures were found in *BCL2A1*^high^ versus *BCL2A1*^low^ samples. **(G)** Spearman correlation between cell-death pathway scores and *BCL2A1*, *GZMB*, and *PRF1* expression.

### Single-cell RNA-seq (GSE176021) discovery of the *BCL2A1*-associated immune phenotype in LUAD patients with on-treatment of ICB

Bulk RNA-seq analysis showed significantly higher CD8^+^ signal in responders within GSE161537 (P = 0.038; **Figure 2A**). UMAP projection from GSE176021 showed *BCL2A1*-expressing CD8^+^ TILs distributed in discrete regions of the CD8^+^ compartment, shown separately for tumors achieving a major pathological response (MPR) and non-MPR tumors (**Figure 2B**). This panel is descriptive and is not used to infer quantitative enrichment in MPR tumors. Pathway-level AUCell analysis at patient level (n = 11 paired) showed that *BCL2A1*^high^ CD8^+^ TILs scored higher on the anti-apoptotic gene set than paired *BCL2A1*^low^ cells in every patient (mean 0.317 vs 0.140; q = 0.004, d_paired = +11.5, 11/11), together with elevated necroptosis (q = 0.004, d_paired = +1.28, 11/11). Because *BCL2A1* is itself a member of that gene set, removing it reduced this difference roughly 12-fold (Cohen’s d +11.5 → +0.9), with the difference abolished in GSE179994 (d = −0.2) and GSE243013 (d = −0.3) and no co-upregulation of the other family members. The elevated score therefore reflects high expression of the anti-apoptotic molecule *BCL2A1* itself rather than coordinated activation of a broader BCL2-family survival program, and we refer to it as a *BCL2A1*-specific transcriptional feature rather than as demonstrated anti-apoptotic pathway activity, which was not measured here. Conversely, cytotoxicity (q = 0.004, d_paired = −1.00) and exhaustion (q = 0.004, d_paired = −1.30) were lower in the *BCL2A1*^high^ fraction (1/11 patients High > Low for each). Apoptosis, ferroptosis, pyroptosis, autophagy, and IFN-γ signalling did not differ after FDR correction (q > 0.05) (paired Wilcoxon, BH-FDR within a 9-pathway family (**Figure 2C**). To complement the limited power of patient-level pathway testing at n = 11, gene-level differential expression was assessed (**Figure 2D**), confirming upregulation of *BCL2A1*, *MCL1*, *TNF*, and *IFNG* and downregulation of cytotoxic (*PRF1*, *GNLY*) and exhaustion (*PDCD1*, *CTLA4*, *LAG3*) genes in the *BCL2A1*^high^ group (all FDR < 0.05). Pseudotime trajectory reconstruction placed *BCL2A1*^high^ cells predominantly in early transitional states (Early Tex/Pre Tex) rather than terminally differentiated or exhausted lineages (7, 8) (**Supplementary Figure S1**). Regulatory analysis indicated that *BCL2A1*^high^ cells are predominantly influenced by *TCF7* and NF-κB family members (*REL*, *NFKB1*, *RELB*, and *RELA*) (18, 19), with additional contributions from *BCL6* (**Supplementary Table S4**), consistent with an early, less-differentiated CD8^+^ T-cell transcriptional state.

**Figure 2:**
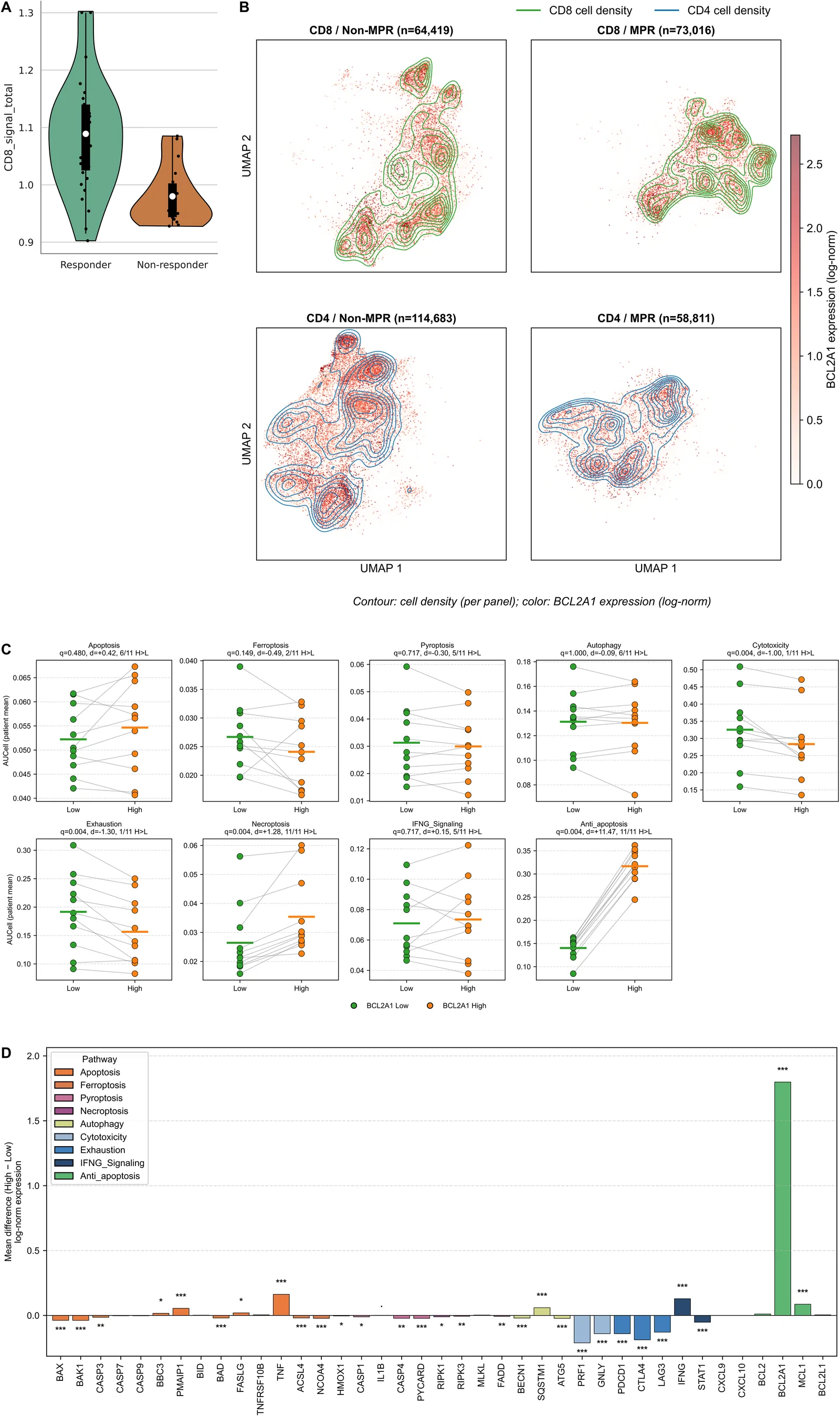
*BCL2A1*-associated immune and cell-death programs in CD8^+^ TILs in GSE176021. **(A)** CD8^+^ T-cell signal (total) in responders versus non-responders in the bulk cohort (GSE161537), estimated by EPIC immune deconvolution. Violin plots with overlaid box-and-whisker; each dot is one patient. **(B)** UMAP of immune cells from GSE176021 coloured by *BCL2A1* expression level (0–4, red = high). Cells from non-MPR tumors (left) and MPR tumors (right) are shown separately. Green and blue contour lines delineate the territories of CD8^+^ and CD4^+^ T cells, respectively. **(C)** AUCell pathway scores in *BCL2A1*-high vs. *BCL2A1*-low CD8 T cells (GSE176021). Paired dot plots of nine pathways across n = 11 paired patients; each patient contributes one point per group (green = Low, orange = High) connected by a grey line, with a horizontal segment at the group mean. Per panel: q (paired Wilcoxon, BH-FDR), Cohen d_paired, and the number of patients with High > Low. **(D)** Differential expression of pathway executioner genes in CD8^+^ TIL profiles grouped by *BCL2A1* level in a single cell cohort (GSE176021). Bars represent mean expression differences between *BCL2A1*^high^ and *BCL2A1*^low^ samples in log-normalized expression, coloured by associated functional pathway. Significance markers: *** q < 0.001, ** < 0.01, * < 0.05, · <0.1 trend; unmarked = not significant.

To characterize the clonal architecture of *BCL2A1*^high^ CD8^+^ TILs in the on-treatment setting, we performed TCR repertoire analysis at the patient level (n = 11; paired Wilcoxon signed-rank, with Cohen’s d_paired as effect size). *BCL2A1*^high^ cells consistently showed a more even, less clonally expanded repertoire than *BCL2A1*^low^ cells. They had lower raw Shannon entropy (6.30 vs. 7.16, d = −2.2; **Supplementary Figure S2A**) and higher clonotype richness (47.7 vs. 22.3 per 100 cells, d = +2.3; **Supplementary Figure S2B**). Raw Shannon entropy is read at very different depths in the two compartments: a median of 309 cells and 147 clonotypes per patient in the *BCL2A1*^high^ compartment against 4,889 cells and 1,300 clonotypes in the *BCL2A1*^low^ compartment. Because the maximum attainable Shannon entropy is constrained by the number of observed clonotypes, the raw comparison is sensitive to that difference. After matching sampling depth within each patient by subsampling the *BCL2A1*^low^ compartment to the corresponding *BCL2A1*^high^ cell count (1,000 iterations), the difference was no longer detectable (6.30 vs. 6.27, P = 0.58). Consistent with this, a lower fraction of *BCL2A1*^high^ cells resided in expanded clones (≥2 cells: 61.5% vs. 83.4%, d = −1.8; ≥3 cells: 52.9% vs. 79.4%, d = −2.4), with a correspondingly higher proportion of single-cell clonotypes (**Supplementary Figures S2C, D**). Repertoire evenness was likewise higher in the *BCL2A1*^high^ compartment (Pielou J′ = 0.89 vs. 0.76, d = +1.5; **Supplementary Figure S2E**). Richness, expanded-clone fractions and evenness were all significant across the 11 patients (P ≤ 2.9×10^-^³). Thus, even at an early treatment time point (two cycles of neoadjuvant therapy), *BCL2A1*^high^ cells constituted a diverse pool of predominantly small clones rather than a few dominant expanded clones.

### Single-cell validation in the longitudinal GSE179994 cohort

In the longitudinal GSE179994 cohort, patient-level paired analysis (pre-treatment n = 18, post-treatment n = 8) confirmed the findings of the discovery cohort. At baseline, *BCL2A1*^high^ CD8^+^ TILs scored higher on the anti-apoptotic gene set than paired *BCL2A1*^low^ cells in all 18 patients (mean 0.221 vs 0.116; paired Wilcoxon q = 6.9×10^-5^, d_paired = +4.88), while exhaustion was lower in the *BCL2A1*^high^ fraction (q = 0.030). Cytotoxicity and necroptosis did not reach significance after FDR correction at baseline (q-values of 0.150 and 0.103, respectively). Gene-level differences confirmed the same direction, with *BCL2A1* and TNF higher in *BCL2A1*^high^ cells and effector (*PRF1*, *GNLY*) and exhaustion (*PDCD1*, *CTLA4*, *LAG3*) genes lower (BH-FDR < 0.05; **Figures 3A, B**). After treatment, the higher anti-apoptotic gene-set score and lower exhaustion score were again observed (anti-apoptosis q = 0.035, d_paired = +3.57, all 8 patients; exhaustion q = 0.035), now accompanied by lower cytotoxicity and ferroptosis (both q = 0.035) in *BCL2A1*^high^ cells (**Figures 3C, D**). Necroptosis showed a non-significant trend at both time points (q = 0.103 pre, 0.070 post). *BCL2A1*^high^ CD8^+^ T cells occupied earlier positions along the pseudotime trajectory than *BCL2A1*^low^ cells, consistent with a less differentiated state (**Figure 4A**). Before treatment, both groups were predominantly composed of Early Tex phenotypes (~94%). However, after treatment, *BCL2A1*^high^ cells largely remained in Early Tex (84.6%), whereas *BCL2A1*^low^ cells shifted toward the more dysfunctional ICB Tex state (20.9%) (**Figures 4B, C**). Thus, the *BCL2A1*^high^ compartment retained an Early Tex–enriched transcriptional distribution at both time points, whereas the *BCL2A1*^low^ compartment shifted toward the ICB Tex state. We interpret this as population-level phenotypic stability rather than persistence of the same cells through therapy: clonal persistence between paired pre- and post-treatment samples did not differ between groups (Jaccard overlap 0.10 vs. 0.12, P = 0.20; fraction of pre-treatment clones persisting 0.30 vs. 0.35, P = 0.31; n = 8).

**Figure 3:**
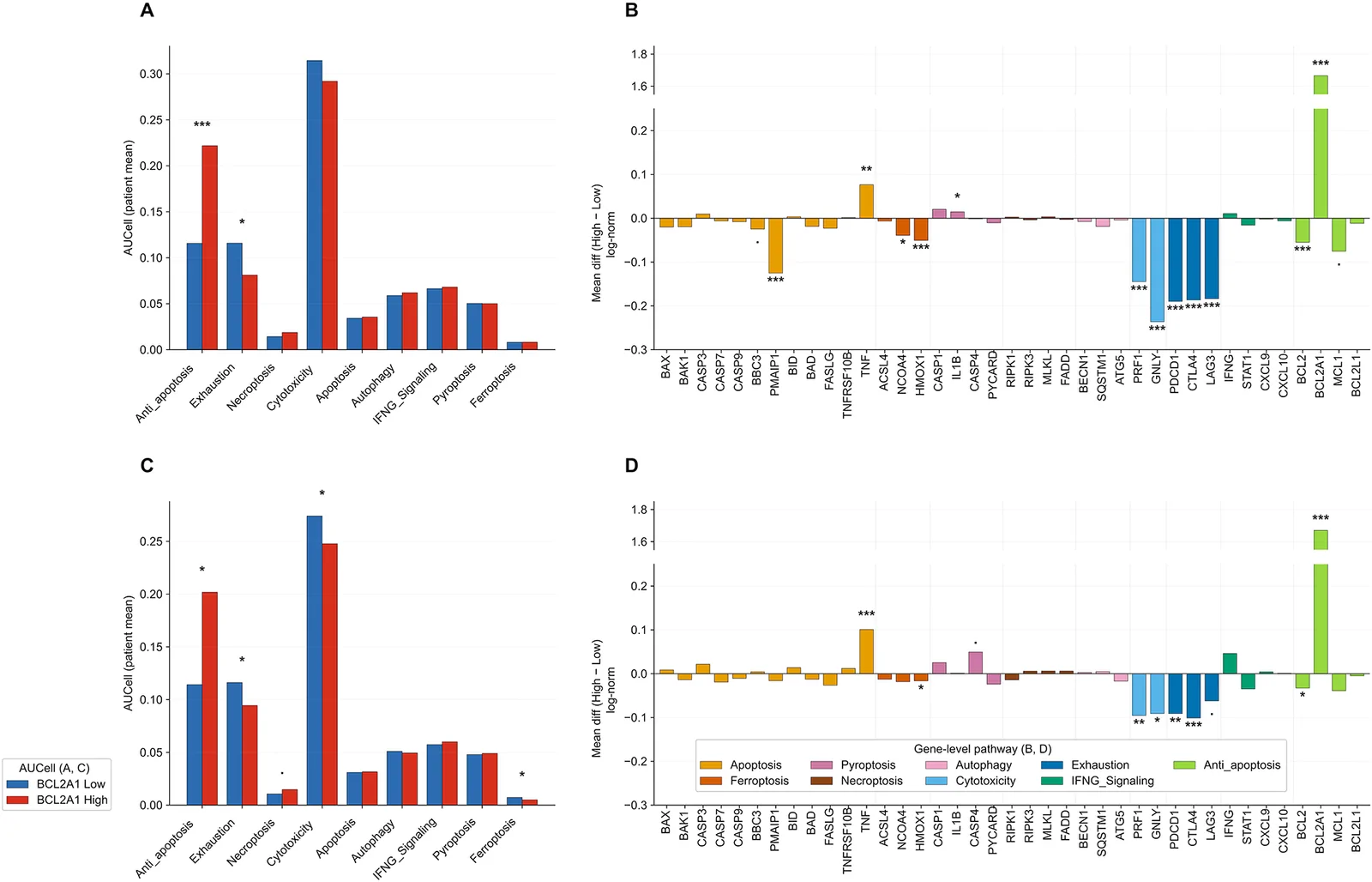
GSE179994 (longitudinal NSCLC): Pathway and gene-level differential expression in *BCL2A1* high vs low CD8 T cells across pre- and post-treatment timepoints. **(A)** AUCell per-patient mean for 9 pathways at PRE-treatment (n = 18 paired patients); bars compare *BCL2A1* Low (blue) vs High (red); significance from paired Wilcoxon with BH-FDR within the 9-pathway timepoint family. **(B)** Gene-level mean difference (High − Low, log-normalized expression) for 39 genes grouped by pathway at PRE-treatment; significance from BH-FDR (Mann–Whitney within timepoint family). **(C)** AUCell per-patient mean for the same 9 pathways at POST-treatment (n = 8 paired patients); BH-FDR applied within POST timepoint family separately from PRE; same conventions as **(A)**. **(D)** Gene-level mean difference for the same 39 genes at POST-treatment; same conventions as **(B)**. Significance: *** q (FDR) < 0.001, ** < 0.01, * < 0.05, · < 0.1 (trend); unmarked = not significant.

**Figure 4:**
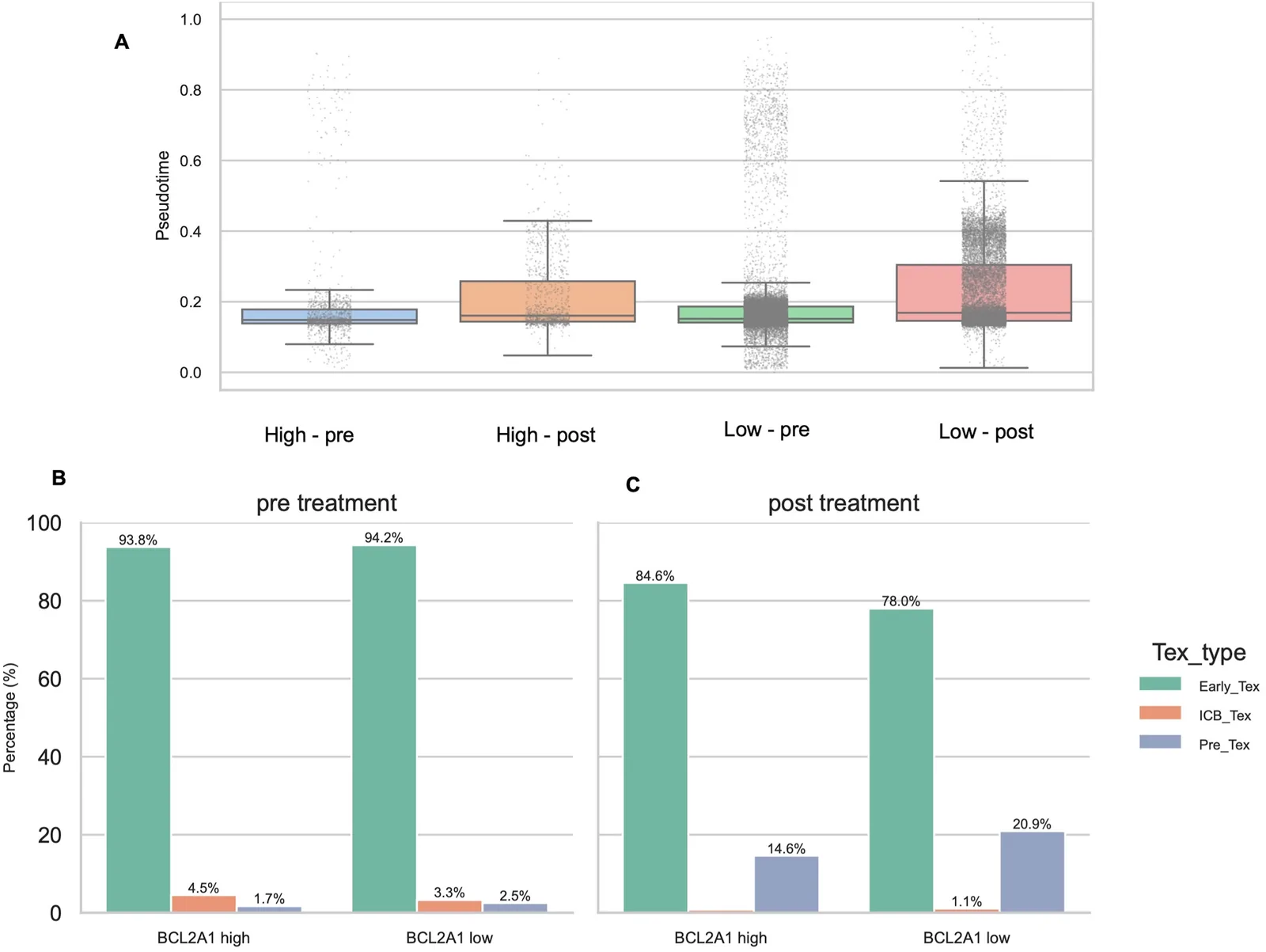
Pseudotime and exhaustion state by the *BCL2A1* group and treatment in paired pre- and post-treatment samples (GSE179994). **(A)** Violin plot of pseudotime values for *BCL2A1*^high^ versus *BCL2A1*^low^ CD8^+^ TILs. Barplot showing distribution of exhaustion phenotypes (Early Tex, Pre Tex, ICB Tex) across *BCL2A1* groups, separated by pre-treatment **(B)** and post-treatment **(C)**.

Regulon analysis distinguished between-group module activity from the underlying regulatory architecture. AUCell activity for the eight BCL2A1-associated regulons (*RELA*, *RELB*, *NFKB1*, *TCF7*, *TOX*, *TBX21*, *EOMES*, *BCL6*) was uniformly high and near-saturated across all groups (~0.95–0.96), with only marginal differences between *BCL2A1*^high^ and *BCL2A1*^low^ cells at either timepoint (**Supplementary Figure S3A**). Within *BCL2A1*^high^ cells, however, *BCL2A1* expression was strongly and monotonically associated with each regulon’s activity both before (ρ = 0.83) and after treatment (ρ = 0.82), consistently across all eight transcription factors (**Supplementary Figures S3B, C**). Thus, while absolute regulon module activity was similar between groups, the regulon architecture co-varying with *BCL2A1* was stable across treatment, consistent with a coordinated NF-κB– and *TCF7/BCL6*-linked program in *BCL2A1*^high^ cells.

Clonal dynamics were re-examined at the patient level (paired Wilcoxon test, Cohen’s d_paired). Contrary to a pattern of preferential expansion, *BCL2A1*^high^ CD8^+^ T cells maintained a less expanded repertoire than paired *BCL2A1*^low^ cells at both timepoints: the proportion of cells in expanded clones (≥2 cells) was lower in *BCL2A1*^high^ cells before treatment (17.3% vs. 54.9%; d_paired = −2.6, P = 3.1×10^-5^, n = 16) and remained lower after treatment (28.1% vs. 68.1%; d_paired = −2.2, P = 7.8×10^-^³, n = 8), lower in every patient at both timepoints (**Figure 5A**). Mean clone size was correspondingly smaller in *BCL2A1*^high^ cells (pre 1.11 vs. 1.78; post 1.22 vs. 2.39; lower in every patient) (**Figure 5B**; **Supplementary Table S5**). Rather than undergoing preferential clonal amplification or preferential persistence, *BCL2A1*^high^ cells remained a diverse pool of small clones across treatment.

**Figure 5:**
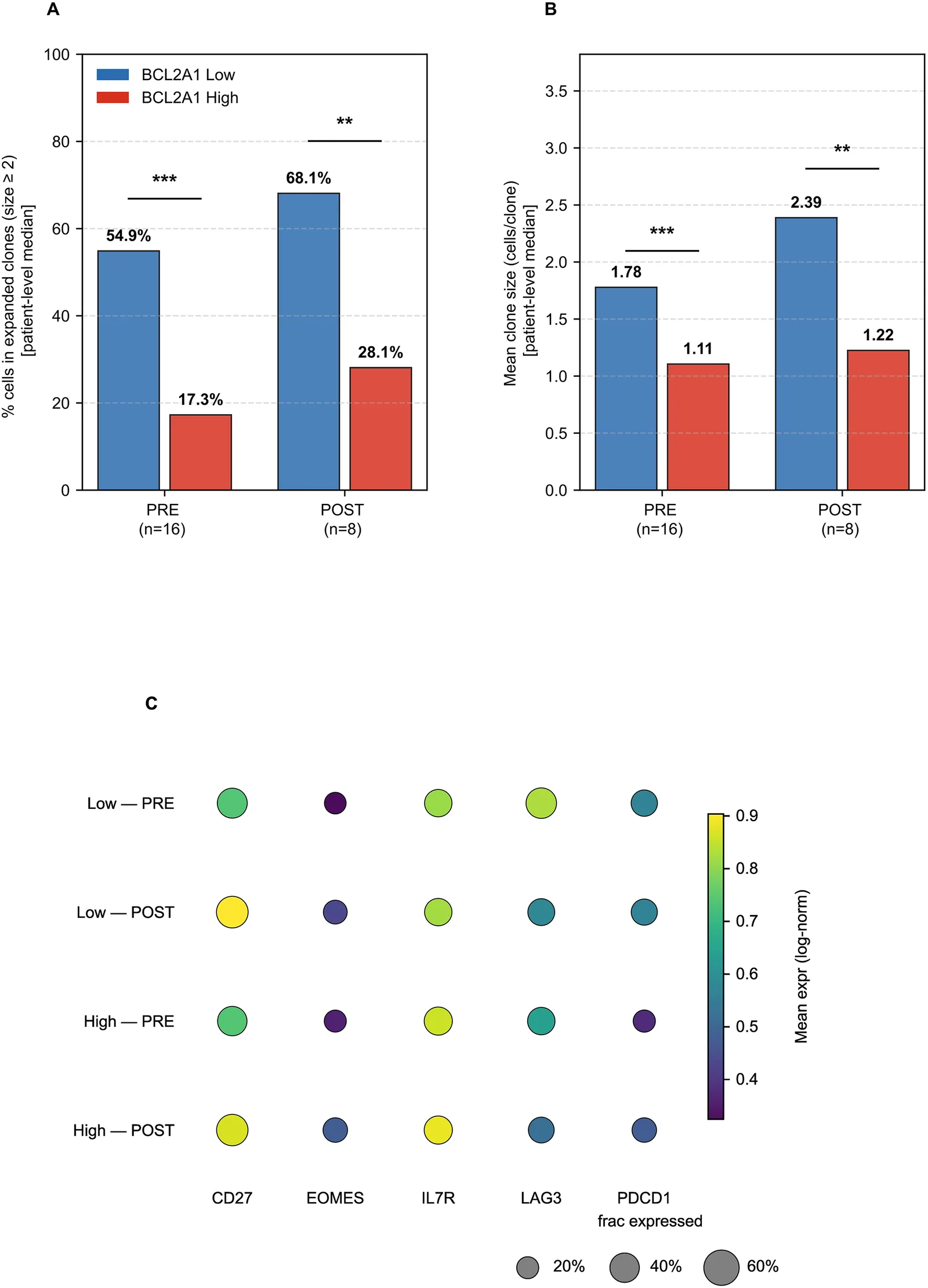
GSE179994 (longitudinal NSCLC): clonal expansion, clone size, and marker expression in *BCL2A1* high vs low CD8 T cells (pre vs post anti-PD-1). Clonal repertoire and marker expression in *BCL2A1* high vs low CD8 T cells from GSE179994 (pre n=16, post n=8 paired patients). **(A)** Percentage of cells in expanded clones (size ≥2) at each time point. **(B)** Mean clone size at each time point. In A and B, grouped bars show patient-level medians (blue = Low, red = High); P values from paired Wilcoxon tests. **(C)** Dotplot of five markers (*CD27*, *EOMES*, *IL7R*, *LAG3*, *PDCD1*) across Low/High × pre/post groups; color = mean expression, size = fraction of expressing cells. Significance: ***P<0.001, **P<0.01.

Phenotypically, post-treatment *BCL2A1*^high^ cells showed lower expression of canonical exhaustion markers (*PDCD1*, *LAG3*) and higher *EOMES*, consistent with a transitional, low-exhaustion profile, whereas the memory markers *CD27* and *IL7R* were comparable between groups (**Figure 5C**).

Together, these observations indicate that the *BCL2A1*^high^ compartment in the longitudinal cohort retained elevated *BCL2A1* expression and reduced exhaustion-associated transcription across treatment, with an Early Tex–enriched distribution and a diverse population of small clones present at both time points.

### Independent validation of the BCL2A1-associated CD8^+^ TIL state

In the independent post-treatment cohort (GSE243013, n = 59), gene-level expression confirmed the same program. *BCL2A1*^high^ CD8^+^ T cells showed the expected specific elevation of *BCL2A1* itself, but no coordinated upregulation of other anti-apoptotic genes: *MCL1*, *BAG3*, *BIRC3*, *FTH1*, and *BCL2* were comparable to or slightly lower than in *BCL2A1*^low^ cells (**Supplementary Figure S4A**), consistent with a *BCL2A1*-specific rather than a global anti-apoptotic shift. Pro-apoptotic genes were not coordinately elevated (**Supplementary Figure S4B**). Exhaustion markers (*PDCD1*, *HAVCR2*, *TOX*, *LAG3*, *TIGIT*, *CTLA4*, *ENTPD1*) were uniformly lower in *BCL2A1*^high^ cells, and cytotoxic-effector genes (*GZMB*, *PRF1*, *GZMA*, *GZMH*, *GNLY*) were also lower, with only IFNG marginally higher (**Supplementary Figure S4C**). Thus, in this independent cohort, *BCL2A1*^high^ CD8^+^ T cells displayed selective *BCL2A1* expression, along with reduced exhaustion and cytotoxic-effector output, matching the findings in the discovery and longitudinal cohorts. Subtype composition differed between groups (patient-level paired test, BH-FDR): *BCL2A1*^high^ CD8^+^ T cells were enriched in effector-memory subtypes (CD8T_Tem_GZMK^+^GZMH^+^ and CD8T_Tem_GZMK^+^NR4A1^+^; both q < 0.001) and depleted in exhausted and terminally differentiated subtypes (CD8T_Tex_CXCL13 q < 2×10^-9^, terminal Tex_LAYN q < 1×10^-4^, proliferating prfh_MKI67 q < 0.01), consistent with a shift away from terminal exhaustion (**Supplementary Figure S5A**). A comparable GZMK^+^ effector-memory CD8^+^ population carrying an anti-apoptotic transcriptional signature has been described independently by single-cell RNA/TCR profiling of patient-derived T cells following CD5-ablated adoptive cell therapy for haematological malignancy (27), a setting distinct from checkpoint blockade in solid tumours. That study also documented robust clonal proliferation within the broader CD5-negative CD8^+^ population, indicating that anti-apoptotic features and clonal proliferation can coexist at the population level and are not obligatorily coupled. Within the *BCL2A1*^high^ compartment, low clonal expansion was observed across the constituent subtypes and was not restricted to any single subtype (**Supplementary Figure S5B**). Regulon analysis distinguished network importance from module activity. In the *BCL2A1*^high^ gene regulatory network, the transcription factors *BCL6*, *NFKB1*, and *TCF7* carried higher regulon importance (GRNBoost2) than in *BCL2A1*^low^ cells, whereas other regulons (*RELB*, *TOX*, *TBX21*, *EOMES*) did not (**Supplementary Figure S6A**). Regulon module activity (AUCell), by contrast, was comparable between groups across all eight transcription factors (**Supplementary Figure S6B**). Thus, although absolute regulon activity was similar, the *BCL2A1*^high^ regulatory architecture preferentially weighted the NF-κB (*NFKB1*), progenitor (*TCF7*), and *BCL6* regulons, consistent with the regulatory pattern seen in the discovery and longitudinal cohorts. TCR profiling showed that *BCL2A1*^high^ CD8^+^ T cells had a more even, less clonally focused repertoire than *BCL2A1*^low^ cells (Pielou J′ = 0.98 vs. 0.89, d_paired = +1.8, n = 56; **Supplementary Figure S7D**), with a lower fraction of cells in expanded clones (≥2 cells: 23.5% vs. 68.8%; ≥3 cells: 10.6% vs. 57.6%; both P < 10^-^¹^6^; **Supplementary Figures S7B, C**), consistent with the diverse, small-clone pattern seen in the discovery and longitudinal cohorts. In summary, *BCL2A1*^high^ CD8^+^ T cells in GSE243013 displayed selective *BCL2A1* expression with reduced exhaustion and reduced cytotoxic-effector output, were enriched in effector-memory subtypes and depleted of exhausted subtypes, preferentially weighted the *BCL6*/*NFKB1*/*TCF7* regulons, and maintained a diverse, low-expansion clonal repertoire — recapitulating the discovery and longitudinal cohorts.

### *In silico* perturbation analysis reveals differential *BCL2A1* dependencies

CellOracle perturbation analysis revealed distinct regulatory sensitivities across transcription factors (22). Knockout (KO) simulations showed NFKB1 and RELA with the highest perturbation magnitudes, whereas overexpression (OE) simulations revealed inverse patterns (**Figures 6A, B**). OE/KO ratio quantification showed *BCL6* (4.47×) and *TCF7* (3.41×) with overexpression-dominant effects, whereas *BCL2A1* exhibited a near-balanced ratio (1.09×), consistent with its role as an anti-apoptotic effector (**Figure 6C**). Cluster-specific analysis revealed that exhausted and terminally differentiated subsets (CD8_Tex_Exhausted, CD8_Terminally_differentiated) exhibited the highest perturbation sensitivity for NF-κB family members and TOX. In contrast, memory-like populations (CD8_Teff_Memory, CD8_Trm_1) showed minimal sensitivity (**Figures 6D, E**). In summary, CellOracle showed strong NF-κB–driven regulatory sensitivity, OE-dominant effects for BCL6 and TCF7, and the highest perturbation vulnerability in exhausted CD8^+^ subsets, with memory-like cells minimally affected.

**Figure 6:**
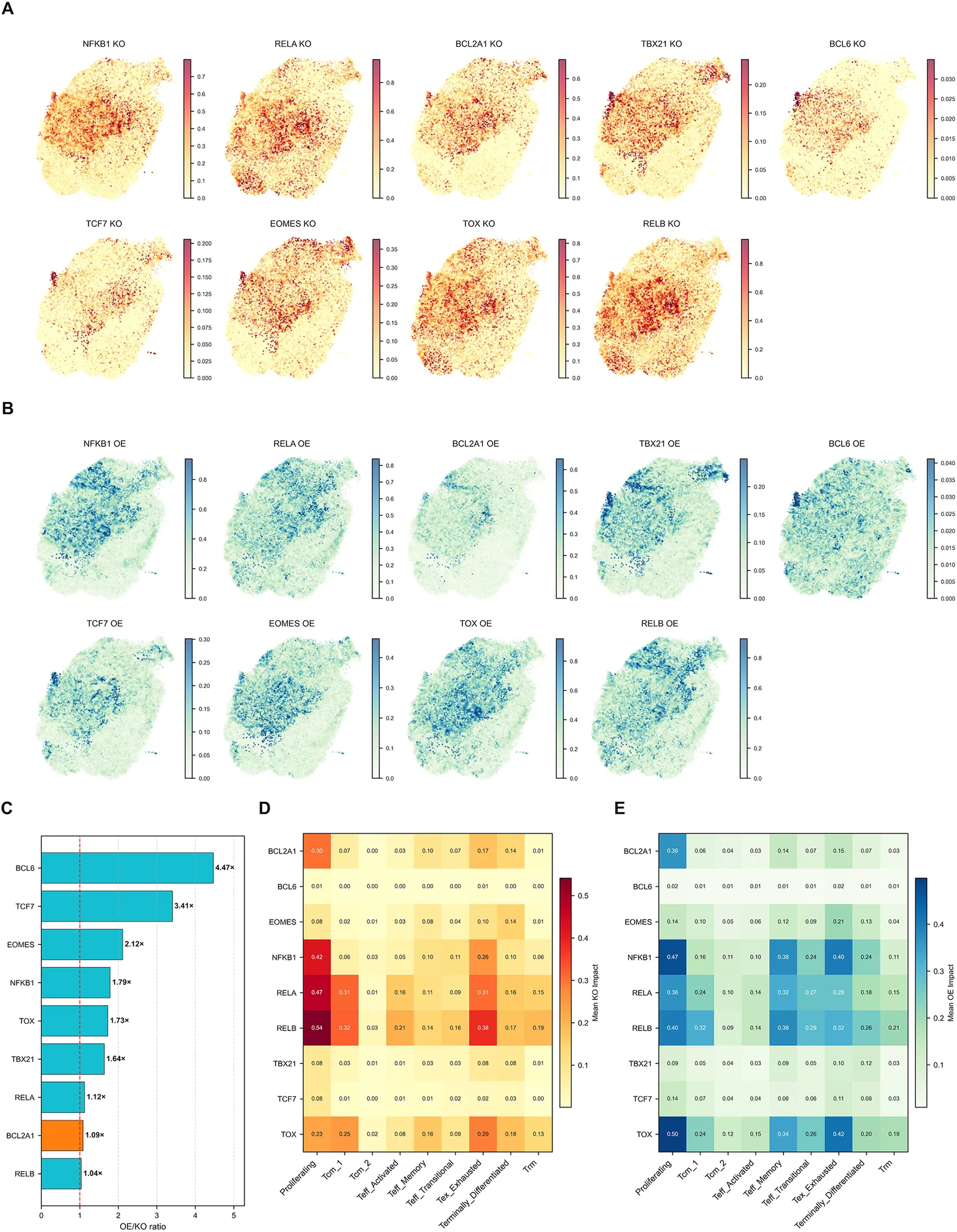
CellOracle knockout and overexpression effects in CD8^+^ T cells (GSE176021). **(A)** Predicted transcription factor knockout **(KO)** perturbation magnitude maps for CD8^+^ T cells. **(B)** Predicted overexpression **(OE)** perturbation magnitude maps for the same regulators. **(C)** Ranked OE/KO effect-size ratio across regulators. **(D)** Heatmap of KO perturbation magnitude by transcription factor and CD8^+^ T-cell subtype. **(E)** Heatmap of OE perturbation magnitude by transcription factor and CD8^+^ T-cell subtype. Perturbation magnitudes are L2 norms of the per-cell expression-shift vector and are therefore non-negative by construction.

### PROGENy pathway analysis identifies elevated NF-κB and TNF-α signaling across cohorts

Using PROGENy pathway analysis (decoupleR) (23, 24) at the patient level, NF-κB emerged as the most strongly and consistently elevated pathway in *BCL2A1*^high^ CD8^+^ T cells across both cohorts. In the discovery cohort (GSE176021), NF-κB activity was higher in *BCL2A1*^high^ cells in all 11 patients (Δ = +0.330, d = +1.89, q = 0.005), and TNF-α activity was similarly elevated (Δ = +0.128, d = +1.06, q = 0.017) (**Figure 7A**). In the longitudinal cohort (GSE179994), both signatures were confirmed before treatment (NF-κB Δ = +0.240, d = +0.79, q = 0.005; TNF-α Δ = +0.183, d = +0.92, q = 0.028) and remained elevated after treatment, at trend level given the smaller post-treatment sample (NF-κB Δ = +0.134, d = +1.85; TNF-α Δ = +0.194, d = +1.81; both q = 0.055, n = 8) (**Figure 7B**). The per-patient paired structure confirmed that the NF-κB difference held in every patient in both cohorts (**Figure 7C**). No other pathway reached significance in both cohorts after correction. NicheNet ligand-activity analysis (nichenetr) nominated TNF as the top-ranked upstream ligand for the *BCL2A1*^high^ transcriptional program (rank 1 of 121 candidate ligands; bootstrap rank 1 in 93 of 100 resamples), with this ranking specific to the *BCL2A1*^high^ gene program (target-gene permutation P = 0.028) (**Figure 7D**). Together with the elevated NF-κB pathway activity, these results nominate a TNF–NF-κB–BCL2A1 axis as a candidate upstream input, which we examine further using the cross-platform TNF-α stimulation data below.

**Figure 7:**
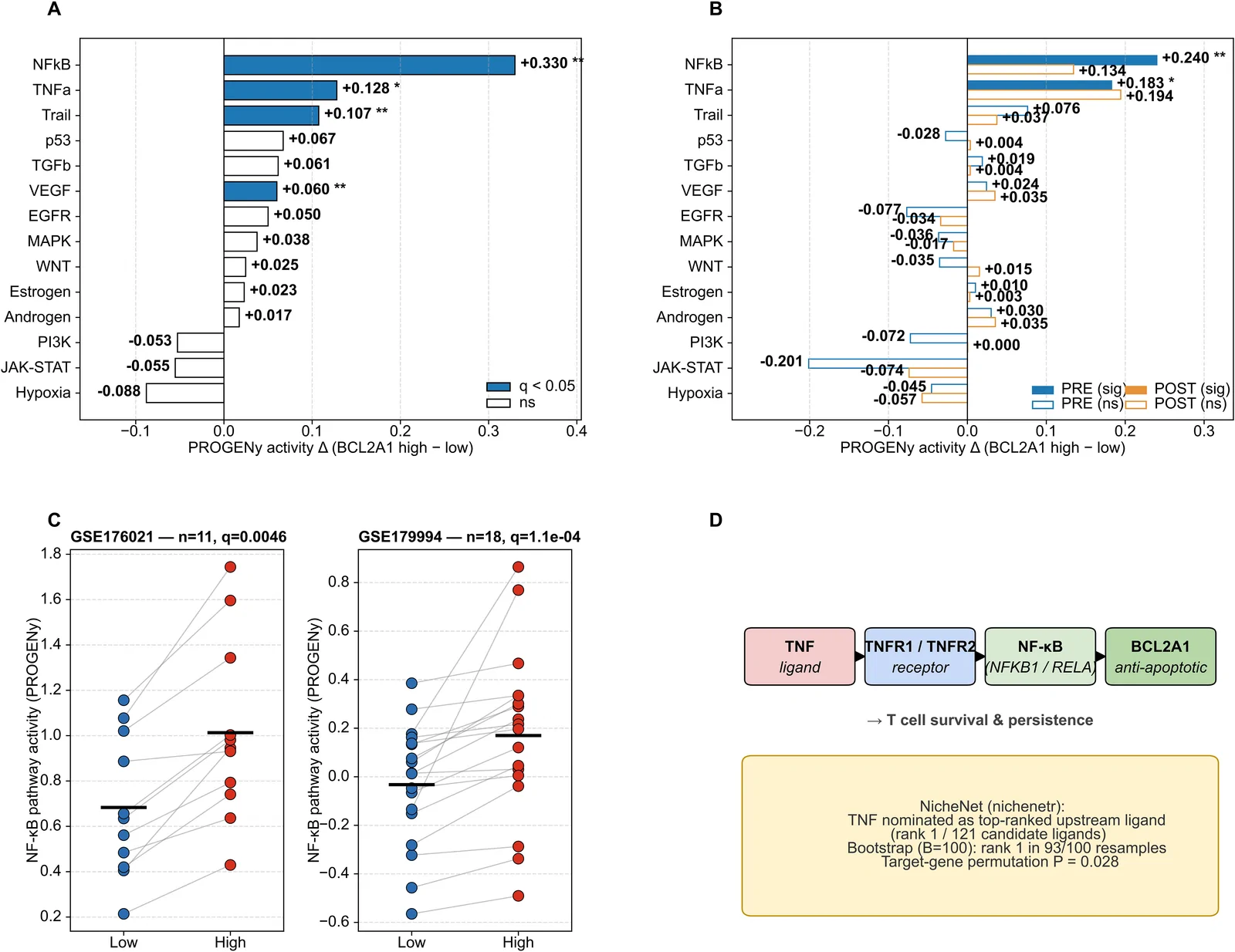
PROGENy pathway activity and NicheNet ligand nomination in *BCL2A1*^high^ CD8^+^ T cells. **(A)** PROGENy pathway activity differences (Δ = *BCL2A1* high − low) across 14 signaling pathways in the discovery cohort (GSE176021), computed at the patient level (n = 11) and tested by paired Wilcoxon signed-rank test with Benjamini–Hochberg correction. Filled bars, q < 0.05; open bars, not significant. *q < 0.05; **q < 0.01 (paired Wilcoxon signed-rank test with Benjamini–Hochberg correction). **(B)** Patient-level pathway activity differences in the longitudinal cohort (GSE179994), shown separately for pre-treatment (n = 18) and post-treatment (n = 8) samples; significance as in **(A)**, corrected within each timepoint. **(C)** Per-patient NF-κB pathway activity in *BCL2A1*^high^ versus *BCL2A1*^low^ cells, with each line connecting one patient and the horizontal dash marking the group mean (GSE176021, n = 11, q = 0.005; GSE179994, n = 18 patients, pooling the available pre- and post-treatment cells within each patient, q = 0.0001). Unlike **(B)**, panel **(C)** does not separate the two timepoints. **(D)** Schematic of the inferred TNF–NF-κB–BCL2A1 cascade. NicheNet ligand-activity analysis (nichenetr) nominated TNF as the top-ranked upstream ligand for the *BCL2A1*^high^ transcriptional program (rank 1 of 121 candidate ligands; bootstrap rank 1 in 93 of 100 resamples; target-gene permutation P = 0.028).

### Selective reduction of biosynthetic flux in *BCL2A1*^high^ CD8^+^ T cells

scFEA flux modeling (26) revealed a selective reduction of biosynthetic flux in *BCL2A1*^high^ CD8^+^ TILs, concentrated in branched-chain amino acid (BCAA) and purine metabolism, rather than a global metabolic shift. Both modules were lower in *BCL2A1*^high^ cells across all three cohorts and both treatment timepoints (directionally consistent in all four strata; **Supplementary Tables S6**–**S8**). BCAA flux was significantly reduced in the discovery cohort (GSE176021: Δ = −4.95×10^-4^, d = −1.14, q = 0.021) and, critically, in the independent validation cohort (GSE243013, n = 57: BCAA q = 0.0012; purine q = 4.7×10^-4^), with both modules also significant post-treatment in GSE179994 (q = 0.043). The purine reduction was independently corroborated by an orthogonal pathway-scoring method (AUCell), which showed the same direction across all cohorts examined. By contrast, central energy modules did not show consistent change, indicating that the *BCL2A1*^high^ state is not globally hypometabolic but rather down-tunes the nucleotide and amino acid biosynthesis required for proliferation—coherent with its diverse, low-expansion TCR repertoire and reduced effector signature.

### Cross-platform validation of TNF-α-induced *BCL2A1* upregulation

To evaluate transcriptional evidence consistent with the candidate TNF-α→NF-κB→BCL2A1 pathway activation (28–31), we analyzed two independent scRNA-seq cohorts with distinct stimulation durations and sequencing platforms. *BCL2A1* was consistently upregulated across both datasets (GSE205095: 1.41-fold in CD8^+^ T cells, p=0.021; GSE289019: 1.33-fold, p=6.13×10^-7^), while classical BCL2 showed no significant response (1.03–1.09×, p=0.549) and *BCL2L2* exhibited discordant patterns between timepoints (**Figure 8A**). Among five *BCL2* family members evaluated, *BCL2A1* showed the highest fold change and strongest statistical significance in TNF-α-stimulated CD8^+^ T cells. Within GSE289019, the TNF-α-responsive CD8^+^ subset showed the highest fold change among the populations examined in that dataset (1.35-fold; 9,219 cells, comprising 4,732 control and 4,487 TNF-stimulated cells), consistent with NF-κB-driven transcriptional induction (**Figure 8B**).

**Figure 8:**
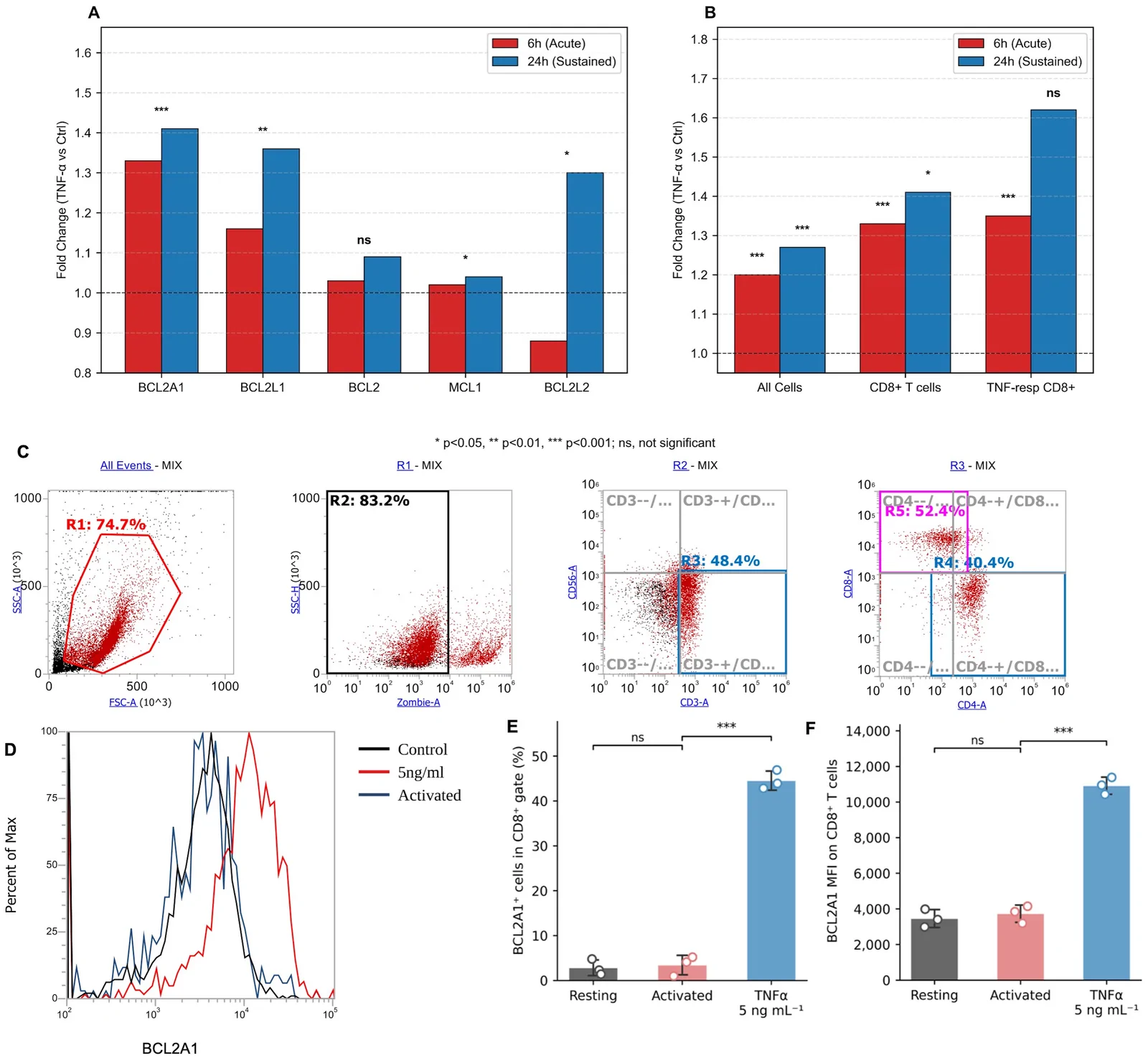
Cross-platform transcriptional evidence and protein-level validation of TNF-α-induced BCL2A1 expression in CD8^+^ T cells. **(A)** BCL2 Family Specificity. Fold change in expression of five BCL2 family members (*BCL2A1*, *BCL2L1, BCL2*, *MCL1*, and *BCL2L2*) in CD8^+^ T cells following TNF-α stimulation at acute (6 h, GSE289019, red) and sustained (24 h, GSE205095, blue) timepoints. **(B)** *BCL2A1* Robustness Across Populations. *BCL2A1* fold change across three hierarchical population strata — all cells, CD8^+^ T cells, and TNF-α-responsive CD8^+^ T cells — at both timepoints, showing consistent upregulation across strata in both datasets. Statistical significance assessed by the Wilcoxon rank-sum test. *p<0.05, **p<0.01, ***p<0.001; ns, not significant. Dashed line at FC = 1.0 represents no change. **(C–F)** Flow cytometry validation in primary human CD8^+^ T cells. **(C)** Gating strategy from a representative TNF-α-treated donor: Lymphocytes → Live (Zombie R718^-^) → CD3^+^CD56^-^ T cells → CD8^+^ T cells (CD4^-^CD8^+^). **(D)** Histogram overlay of BCL2A1 fluorescence on gated CD8^+^ T cells from one representative donor; Resting (black) and Activated (blue) overlap, whereas TNF-α (red) is shifted toward higher BCL2A1. **(E)** Percentage of BCL2A1^+^ cells within the CD8^+^ gate (n = 3 donors; mean ± SD). One-way ANOVA F (2,6) = 416.41, P = 3.7 × 10^-7^. **(F)** BCL2A1 MFI on the CD8^+^ population (n = 3 donors; mean ± SD). One-way ANOVA F (2,6) = 223.78, P = 2.3 × 10^-6^. Tukey HSD: ***P < 0.001; ns, P ≥ 0.05. The Wilcoxon rank-sum test was used to assess statistical significance in **(A)**.

### Protein-level validation of TNF-α-induced BCL2A1 in primary CD8^+^ T cells

To test the protein-level prediction of this candidate axis, we measured BCL2A1 protein in primary human CD8^+^ T cells by flow cytometry. Following a standard gating hierarchy (**Figure 8C**), histogram overlays showed a clear rightward shift of the entire TNF-α-treated CD8^+^ distribution relative to Resting and Activated cells, which largely overlapped (**Figure 8D**). Across three independent donors, TNF-α (5 ng mL^-^¹) markedly induced BCL2A1: the proportion of BCL2A1^+^ cells within the CD8^+^ gate rose from 3.43 ± 2.18% in Activated controls to 44.53 ± 2.12% (≈13-fold; Tukey P < 0.0001; **Figure 8E**), and BCL2A1 MFI rose concordantly from 3,733 ± 487 to 10,920 ± 480 (2.93-fold; Tukey P < 0.0001; **Figure 8F**). Critically, anti-CD3/CD28 activation alone did not increase BCL2A1 above the resting baseline by either endpoint (Resting vs. Activated, %BCL2A1^+^ P = 0.93; MFI P = 0.78), indicating that TCR engagement per se is insufficient and that the response is attributable to TNF-α signalling rather than background activation (**Supplementary Tables S9**, **S10**). These data establish TNF-α-induced BCL2A1 protein expression as a measurable response in primary human CD8^+^ T cells; the NF-κB intermediate was not tested and the full axis remains inferred.

## Discussion

Resistance to ICB in NSCLC is often driven by the loss of functional CD8^+^ T-cell responses (32, 33). We previously published evidence supporting the association between BCL2A1high CD8^+^ TILs and response to ICB in NSCLC (15). In this study, we characterised a transcriptionally distinct subset of *BCL2A1*^high^ CD8^+^ TILs marked by an earlier, less-exhausted differentiation state with restrained terminal-effector transcription, reproducibly present across cohorts and across treatment. Through integrated multi-omic analysis across four independent cohorts, we demonstrate consistent enrichment of *BCL2A1*^high^ CD8^+^ TILs in tumors with immune-inflamed features, identifying a cellular correlate of ICB responsiveness in NSCLC.

It is worth stating precisely what was established previously, what is new here, and what remains a model. Our earlier study established the association between *BCL2A1* and *PD-1* blockade response in lung adenocarcinoma, its enrichment in immune-inflamed tumours, and its origin in the immune rather than the tumour compartment (15). The present study newly demonstrates that *BCL2A1*^high^ CD8^+^ TILs occupy a reproducible transcriptional state across four independent cohorts — earlier, less-exhausted differentiation with restrained terminal-effector transcription; that the elevated anti-apoptotic score is BCL2A1-specific rather than BCL2-family-wide; and that TNF-α induces BCL2A1 protein in primary human CD8^+^ T cells. What remains a model rather than a demonstrated mechanism is the functional claim: that BCL2A1 expression confers resistance to activation-induced cell death, and that NF-κB dependence links TNF-α to BCL2A1 in this setting. Neither was tested here; the former requires perturbation of BCL2A1 with a survival endpoint, the latter a TNF-α ± NF-κB inhibition experiment with BCL2A1 protein as the readout.

Even before treatment, bulk *BCL2A1*^high^ tumours displayed concurrent activation and apoptotic stress: increased expression of cytotoxic molecules (*GZMB, PRF1*, and *IFNG*) and death pathway components (*TNF, FASLG*, and *BID*), alongside strong upregulation of anti-apoptotic BCL2A1. Within the CD8^+^ compartment at single-cell resolution, by contrast, terminal effector genes were lower in *BCL2A1*^high^ cells (*PRF1* and *GNLY* in GSE176021; *GZMB*, *PRF1, GZMA, GZMH* and *GNLY* in GSE243013); these observations arise at different levels of resolution and should not be conflated, since bulk tissue aggregates all infiltrating populations whereas single-cell analysis isolates the CD8^+^ compartment. *BCL2A1* is a highly regulated NF-κB target gene that exerts essential pro-survival functions by inhibiting pro-apoptotic BCL2 family members (11, 34). The specificity of this induction is noteworthy: among *BCL2* family members, *BCL2A1* — but not classical *BCL2*, *MCL1*, or *BCL2L2* — consistently responded to TNF-α stimulation in CD8^+^ T cells across independent experimental platforms, suggesting that NF-κB signaling converges preferentially on *BCL2A1* rather than on redundant family members. This selectivity is consistent with the transient, inducible nature of *BCL2A1* transcription. Consistent with this transcriptomic specificity, flow cytometry in primary human CD8^+^ T cells confirmed that TNF-α induces BCL2A1 at the protein level (≈13-fold higher proportion of BCL2A1^+^ cells; 2.93-fold higher median fluorescence intensity; P < 0.0001), whereas anti-CD3/CD28 activation alone did not, indicating that this induction reflects TNF-α signaling rather than generic T-cell activation. This provides direct, protein-level support in primary human cells for one key prediction of the computational model, while the NF-κB intermediate and the downstream survival consequence remain to be tested directly. This co-activation was accompanied by enhanced pyroptosis signaling through inflammatory caspases (*CASP1* and *CASP4*) and adaptor proteins (*IL1B* and *PYCARD*), suggesting that *BCL2A1*^high^ T cells reside in inflammatory niches under intense immunologic stress. Notably, despite expression of some exhaustion-related genes, these cells exhibited significantly lower exhaustion scores, consistent with an earlier differentiation state rather than terminal dysfunction.

In support of this model, TCR repertoire analyses across all three single-cell cohorts revealed a consistent clonal architecture for *BCL2A1*^high^ CD8^+^ TILs: a more even, less clonally expanded repertoire than paired *BCL2A1*^low^ cells. In the on-treatment discovery cohort (GSE176021), *BCL2A1*^high^ cells showed a smaller fraction of cells in expanded clones (≥2 cells: 61.5% vs. 83.4%; patient-level paired Wilcoxon, d = −1.8), indicating a repertoire composed of many small clonotypes rather than a few large ones. The same pattern held in the longitudinal cohort (GSE179994), where the expanded-clone fraction remained lower in *BCL2A1*^high^ cells both before and after treatment (pre 17.3% vs. 54.9%; post 28.1% vs. 68.1%; lower in every patient), and in the independent post-treatment cohort (GSE243013, 23.5% vs. 68.8%). Rather than undergoing preferential clonal amplification, *BCL2A1*^high^ cells maintained a diverse, low-expansion repertoire across timepoints and cohorts. Anti-apoptotic capacity does not in itself imply restricted clonal expansion: in an independent human adoptive-therapy setting, a CD8^+^ population showing both anti-apoptotic transcriptional features and robust clonal proliferation has been described (27). This non-expanding profile is internally coherent with their reduced cytotoxic-effector output and reduced biosynthetic flux (below): cells that are not proliferating into large effector clones would not be expected to dominate the repertoire. Because *BCL2A1*^high^ cells constitute a rare (~5%) subset, count-sensitive diversity indices are necessarily read at fewer cells per patient than in the *BCL2A1*^low^ compartment; the expansion-fraction and evenness measures, which are far less sensitive to sampling depth, are concordant across all cohorts and anchor this conclusion.

The biological interpretation of this clonal architecture should remain open. A diverse, low-expansion repertoire is compatible with a survival-competent reservoir, but it does not by itself establish enhanced survival, and alternative explanations cannot be excluded from these datasets. Reduced clonal expansion may equally reflect lower antigen engagement, differences in tumour-antigen specificity, or simply a less proliferative state — the last being consistent with our own observation that *BCL2A1*^high^ cells show reduced cytotoxic-effector transcription and reduced biosynthetic flux. We therefore report the clonal architecture as a descriptive finding and treat its interpretation as a memory-like reservoir, as evidence of superior survival, or as immune resilience, as hypotheses requiring direct testing.

Notably, while pre-treatment both *BCL2A1*^high^ and *BCL2A1*^low^ groups showed similar Early Tex composition (~94%), post-treatment divergence was striking: *BCL2A1*^high^ cells largely remained in Early Tex (84.6%), whereas *BCL2A1*^low^ cells shifted toward the more dysfunctional ICB Tex state (20.9%). Subtype mapping further revealed that *BCL2A1*^high^ cells are distributed across transitional, Trm-like, and memory-like states rather than restricted to terminally exhausted compartments, suggesting that an earlier differentiation state can be uncoupled from exhaustion when BCL2A1 is highly expressed. Unlike lineage-restricted markers, BCL2A1 expression represents a stress-inducible transcriptional feature predominantly enriched within the Early Tex/progenitor-like compartment—a transcriptional rather than lineage-based definition that resembles the TCF1+ stem-like paradigm (7, 35–37) and may confer superior predictive value by spanning multiple differentiation states (38, 39).

An apparent divergence merits comment: cytotoxic and exhaustion signatures were elevated in *BCL2A1*^high^ tumors in bulk yet reduced within *BCL2A1*^high^ CD8^+^ T cells at single-cell resolution — complementary rather than contradictory, as bulk aggregates all infiltrating populations, whereas single-cell isolates the CD8^+^ compartment. Consistent with this, the bulk signals persisted after adjustment for CD8 abundance by three independent strategies (CD8 fraction explaining ~7% of gene-level variance), indicating that *BCL2A1*^high^ CD8^+^ T cells are not the sole source of the tissue-level signal.

Together, these findings support a model in which *BCL2A1*^high^ CD8^+^ TILs occupy a transcriptional state positioned between effector activation and terminal exhaustion. Under ICB, reinvigorated T cells face high proliferative and inflammatory pressure-often resulting in apoptosis or dysfunction (10, 40). However, *BCL2A1* expression may permit a subset to remain in an earlier, less-exhausted state — a model these data support but do not establish. The transcription factor *TOX* has been identified as a central regulator of exhausted T cells (41, 42), and our findings raise the possibility that *BCL2A1* expression is inversely related to TOX-driven exhaustion programs. We propose the term “buffer-survivor” as a conceptual model for this BCL2A1-associated state, rather than as a functionally established cell state; it may represent a previously underappreciated checkpoint in CD8^+^ T-cell differentiation, distinct from progenitor or terminal Tex subsets.

These mechanistic findings corroborate and extend our previous study, which established *BCL2A1* as a predictive biomarker within a tri-axis framework integrating *BCL2A1*, *CD274 (PD-L1)*, and the HOT score (discovery AUC = 0.826; validation macro-AUC = 0.774, outperforming established signatures including TIDE, TIS, IPS, and IFNG). Several key observations from that work are now mechanistically clarified: the strong *BCL2A1*-Hot_score correlation (*r* = 0.622) reflects enrichment in inflammatory niches with concurrent cytotoxic and pyroptotic signaling; the immune-compartment origin of *BCL2A1* signals is supported by single-cell enrichment specifically in Trm, proliferating, and memory-like subsets rather than tumor cells.

This study has limitations. Our experimental validation was performed in primary human CD8^+^ T cells but was limited to confirming TNF-α-induced *BCL2A1* expression at the protein level (three donors); it did not test the anti-apoptotic function of *BCL2A1*, which should be confirmed through apoptotic challenge with *BCL2A1* knockdown or overexpression in primary CD8+ T cells (43). The TNF-α→BCL2A1 induction was demonstrated in peripheral and non-tumor CD8^+^ T cells (PBMC-derived cross-platform datasets and healthy-donor flow cytometry) rather than in tumor-infiltrating lymphocytes directly. However, NF-κB-driven BCL2A1 induction is a canonical, cell-intrinsic response, but confirmation in CD8^+^ TILs within the tumor microenvironment remains to be established. Inferred regulons are based on co-expression and motif enrichment rather than direct transcriptional evidence; experimental confirmation via ChIP-seq or ATAC-seq is needed (44). The NF-κB intermediate of the TNF-α→BCL2A1 axis remains inferred rather than directly established, and pharmacological inhibition (e.g., Bay 11-7082) is planned to test it. The focus on lung adenocarcinoma limits generalizability to other cancer types. In summary, we define a CD8^+^ TIL state marked by *BCL2A1*, characterised by an earlier, less-exhausted differentiation profile and restrained terminal-effector output. This state lies between effector activation and terminal exhaustion, and its relationship to immune resilience under ICB remains to be tested. Future studies targeting the BCL2A1-NF-κB axis may uncover strategies to enhance antitumor immunity in NSCLC and beyond (45, 46).

## Conclusion

In summary, we identify *BCL2A1*^high^ CD8^+^ TILs as a reproducible transcriptional state marked by lower exhaustion-associated transcription, an earlier differentiation profile, and restrained terminal-effector output, associated with response to ICB in NSCLC. TNF-α induces BCL2A1 protein in primary human CD8^+^ T cells; the NF-κB intermediate and the proposed link to resistance to activation-induced cell death remain inferred and require direct functional testing. These findings characterise BCL2A1 as a marker of this state and identify the TNF-α/BCL2A1 axis as a candidate target for further investigation.

## Data Availability

The original contributions presented in the study are included in the article/**Supplementary Material**. Further inquiries can be directed to the corresponding author.

## Glossary

AICD: activation-induced cell death
BCAA: branched-chain amino acid
BCL2A1: B-cell lymphoma 2-related protein A1
BH: Benjamini–Hochberg
CDR3: complementarity-determining region 3
DPT: diffusion pseudotime
EPIC: estimating the proportions of immune and cancer cells
FDR: false discovery rate
HSD: honestly significant difference
ICB: immune checkpoint blockade
IFN-γ: interferon-γ
IPS: immunophenoscore
KO: knockout
LUAD: lung adenocarcinoma
MFI: median fluorescence intensity
MPR: major pathological response
NF-κB: nuclear factor κB
NK: natural killer
NSCLC: non-small cell lung cancer
OE: overexpression
PBMC: peripheral blood mononuclear cell
PD-1/PD-L1: programmed cell death protein 1/programmed death-ligand 1
P-Tex: proliferative exhausted T cell
scRNA-seq: single-cell RNA sequencing
ssGSEA: single-sample gene set enrichment analysis
TCA: tricarboxylic acid
TCR: T-cell receptor
TCR-seq: T-cell receptor sequencing
TF: transcription factor
Tem: effector memory T cell
Tex: exhausted T cell
Texterm: terminally exhausted T cell
TIDE: Tumor Immune Dysfunction and Exclusion
TIL: tumor-infiltrating lymphocyte
TIS: Tumor Inflammation Signature
Tm: memory T cell
Tpex: precursor exhausted T cell
Trm: tissue-resident memory T cell
UMAP: uniform manifold approximation and projection
VDJ: variable, diversity, and joining
