# Supplementary material for: BCL2A1 marks an early, less-exhausted CD8+ tumor-infiltrating lymphocyte state associated with PD-1 blockade response in NSCLC
Source: Front Immunol. 2026 Oct 7;17:1908216. doi: 10.3389/fimmu.2026.1908216 (PMC13683672; doi:10.3389/fimmu.2026.1908216)
Supplement: Supplementary file 1 [file Table1.docx]

**Supplementary Materials**

**Table S1. Summary of datasets used in this study**. Brief overview of the four transcriptomic cohorts analyzed, including data type, treatment context, sample size, and each dataset’s specific role in evaluating the *BCL2A1-*associated CD8⁺ TIL state.

| **Dataset** | **Data Type** | **Patients/Samples** | **Treatment Context** | **Stage / Notes** | **Role in Hypothesis** |
| --- | --- | --- | --- | --- | --- |
| GSE161537  (n = 60) | Bulk RNA-seq (EPIC to define CD8) | 60 LUAD (pre-treatment, 34 R / 26 NR) | ICB monotherapy | Stage III-IV | Contextual profiling of *BCL2A1*- overview of real-world tumor immunity |
| GSE176021  (n = 11) | scRNA-seq  + TCR | 11 LUAD (44 samples on-treatment, 137,435 CD8⁺ T cells) | Nivolumab, after 2 cycles | Stage I–III | Discovery phase — BCL2A1-associated CD8⁺ TIL state |
| GSE179994  (n = 18) | scRNA-seq + TCR | 18 LUAD (29 samples, 48,253 CD8⁺ T cells) | ICB + chemotherapy | Stage III–IV | Supportive evidence across pseudotime and regulators |
| GSE243013  (n = 59) | scRNA-seq + TCR | 59 LUAD (59 samples post-treatment, 68,181 CD8⁺ T cells) | ICB + chemotherapy (3–15 cycles) | Stage I–IV | Validation using transcriptional and clonal readouts |

**Table S2. Pathway executioner gene expression in the bulk cohort (GSE161537) by *BCL2A1* level.** Per-gene comparison of mean expression between *BCL2A1*^high^ and *BCL2A1*^low^ samples (median split of BCL2A1 log2(CPM+1), n = 30/30), across cell-death, cytotoxic, exhaustion, IFN-γ, and anti-apoptotic pathways. Values are mean log2(CPM+1); significance by Mann–Whitney U with Benjamini–Hochberg correction—genes ordered by pathway.

| **Pathway** | **Gene** | **mean_high** | **mean_low** | **log2fcHL** | **p_value** | **q_BH** | **sig_q005** |
| --- | --- | --- | --- | --- | --- | --- | --- |
| Apoptosis | *BAX* | 7.956617 | 7.8142 | 0.142417 | 0.200949 | 0.285218 | FALSE |
|  | *BAK1* | 8.188626 | 7.827095 | 0.361531 | 0.000284 | 0.000781 | TRUE |
|  | *CASP3* | 8.405386 | 7.823263 | 0.582123 | 8.29E-06 | 7.85E-05 | TRUE |
|  | *CASP7* | 7.872077 | 7.465758 | 0.406319 | 0.003034 | 0.006068 | TRUE |
|  | *CASP9* | 5.429617 | 4.723399 | 0.706217 | 0.020681 | 0.032498 | TRUE |
|  | *DIABLO* | 7.110511 | 7.012705 | 0.097806 | 0.290472 | 0.387296 | FALSE |
|  | *BBC3* | 6.195585 | 6.20371 | -0.00813 | 0.864994 | 0.88511 | FALSE |
|  | *PMAIP1* | 6.818961 | 6.767548 | 0.051413 | 0.807275 | 0.857734 | FALSE |
|  | *BCL2L11* | 6.322087 | 5.867704 | 0.454384 | 0.002755 | 0.005772 | TRUE |
|  | *BID* | 6.990696 | 6.313345 | 0.677351 | 0.000213 | 0.000624 | TRUE |
|  | *BAD* | 7.514183 | 7.451171 | 0.063013 | 0.270705 | 0.37222 | FALSE |
|  | *FASLG* | 5.80246 | 4.893893 | 0.908567 | 0.000201 | 0.000624 | TRUE |
|  | *TNFRSF10B* | 8.557773 | 8.620284 | -0.06251 | 0.818746 | 0.857734 | FALSE |
|  | *TNF* | 5.985246 | 4.875832 | 1.109415 | 2.6E-05 | 0.000127 | TRUE |
| Ferroptosis | *ACSL4* | 8.951169 | 8.629841 | 0.321328 | 0.016285 | 0.026538 | TRUE |
|  | *SLC7A11* | 5.476056 | 5.981923 | -0.50587 | 0.395267 | 0.496907 | FALSE |
|  | *NCOA4* | 7.669201 | 7.660537 | 0.008664 | 0.641424 | 0.783962 | FALSE |
|  | *HMOX1* | 7.867993 | 7.155823 | 0.71217 | 0.000399 | 0.000975 | TRUE |
| Pyroptosis | *CASP1* | 8.497642 | 7.463418 | 1.034224 | 5.53E-08 | 8.11E-07 | TRUE |
|  | *IL1B* | 6.493908 | 5.365134 | 1.128774 | 2.43E-05 | 0.000127 | TRUE |
|  | *CASP4* | 8.185313 | 7.794312 | 0.391001 | 0.013272 | 0.02246 | TRUE |
|  | *PYCARD* | 7.653701 | 6.680011 | 0.973691 | 1.17E-05 | 7.85E-05 | TRUE |
| Autophagy | *BECN1* | 6.358014 | 6.231057 | 0.126957 | 0.761828 | 0.838011 | FALSE |
|  | *SQSTM1* | 10.9538 | 11.09685 | -0.14305 | 0.673495 | 0.800913 | FALSE |
|  | *ATG5* | 7.681988 | 7.493053 | 0.188935 | 0.05012 | 0.076045 | FALSE |
|  | *ULK1* | 8.078816 | 8.126023 | -0.04721 | 0.923442 | 0.923442 | FALSE |
| Cytotoxicity | *GZMB* | 7.343932 | 6.173781 | 1.170152 | 0.000356 | 0.000922 | TRUE |
|  | *PRF1* | 7.064123 | 5.8437 | 1.220423 | 1.25E-05 | 7.85E-05 | TRUE |
|  | *GNLY* | 7.273204 | 6.470001 | 0.803202 | 0.011711 | 0.020611 | TRUE |
|  | *GZMA* | 6.611696 | 5.463572 | 1.148125 | 8.66E-05 | 0.000318 | TRUE |
| Exhaustion | *PDCD1* | 5.927868 | 4.488029 | 1.43984 | 5.09E-08 | 8.11E-07 | TRUE |
|  | *CTLA4* | 6.264598 | 5.309856 | 0.954742 | 8.15E-05 | 0.000318 | TRUE |
|  | *LAG3* | 6.689258 | 5.518269 | 1.170989 | 0.000117 | 0.000398 | TRUE |
| IFNG_Signaling | *IFNG* | 5.990071 | 5.153218 | 0.836853 | 0.000422 | 0.000977 | TRUE |
|  | *STAT1* | 10.60775 | 9.987527 | 0.620223 | 0.006972 | 0.012783 | TRUE |
|  | *CXCL9* | 9.336236 | 7.642565 | 1.693671 | 0.001302 | 0.002864 | TRUE |
|  | *CXCL10* | 8.455825 | 7.35656 | 1.099265 | 0.004427 | 0.008469 | TRUE |
| Anti_apoptosis | *BCL2* | 6.994536 | 5.819836 | 1.1747 | 1.17E-05 | 7.85E-05 | TRUE |
|  | *BCL2A1* | 8.12059 | 6.562641 | 1.55795 | 3.02E-11 | 1.33E-09 | TRUE |
|  | *MCL1* | 10.98904 | 10.77365 | 0.215391 | 0.077272 | 0.113332 | FALSE |
|  | *BCL2L1* | 8.479537 | 8.492887 | -0.01335 | 0.717189 | 0.809136 | FALSE |
| Necroptosis | *RIPK1* | 7.340897 | 7.279912 | 0.060985 | 0.695215 | 0.804986 | FALSE |
|  | *RIPK3* | 6.662816 | 5.762243 | 0.900573 | 8.15E-05 | 0.000318 | TRUE |
|  | *TNF* | 5.985246 | 4.875832 | 1.109415 | 2.6E-05 | 0.000127 | TRUE |
|  | *FADD* | 8.485281 | 8.356305 | 0.128976 | 0.363222 | 0.470052 | FALSE |

**Table S3. Sensitivity of bulk cytotoxic and exhaustion signals to CD8 T-cell composition.** *BCL2A1* high-versus-low difference in cytotoxicity (*GZMB*, *PRF1*, *GNLY*, *GZMA*) and exhaustion (*PDCD1*, *CTLA4*, *LAG3*) scores — mean log₂(CPM+1) per gene-set — before and after adjustment for the EPIC-estimated CD8 fraction by three methods: (i) CD8 fraction as an OLS covariate; (ii) residualization against CD8 fraction; (iii) down-weighting by (1 − CD8 fraction) in linear space. The raw effect (unadjusted high−low difference) is common to all three. Analysis on n = 51 samples with a non-zero CD8 estimate (*BCL2A1* high = 26, low = 25; median split on *BCL2A1*). P: Mann–Whitney U (ii–iii) or regression t-test (i), two-sided.

| **Outcome** | **Adjustment method** | **Effect (raw)** | **Effect (adj.)** | **Δ** | ***P* (adj.)** |
| --- | --- | --- | --- | --- | --- |
| Cytotoxicity | CD8 covariate | +1.03 | +0.96 | −7.6% | 4.7 × 10⁻⁴ |
|  | Residualization | +1.03 | +0.92 | −10.6% | 7.7 × 10⁻⁴ |
|  | Non-CD8 down-weighting | +1.03 | +0.97 | −6.4% | 4.4 × 10⁻⁴ |
| Exhaustion | CD8 covariate | +1.16 | +1.10 | −5.1% | 1.5 × 10⁻⁶ |
|  | Residualization | +1.16 | +1.07 | −8.2% | 9.9 × 10⁻⁶ |
|  | Non-CD8 down-weighting | +1.16 | +1.09 | −6.2% | 7.6 × 10⁻⁶ |

**Table S4. Predicted upstream transcription factors regulating *BCL2A1* identified by SCENIC in on-treatment single-cell RNA-seq cohort.** Transcription factor–target gene relationships were inferred using the SCENIC pipeline. Gene regulatory networks were constructed with GRNBoost2, followed by motif enrichment–based pruning using RcisTarget to retain direct TF–target associations. The table includes transcription factors with *BCL2A1* as a predicted target, along with their importance scores as computed by GRNBoost2.

| TF | target | importance |
| --- | --- | --- |
| REL | BCL2A1 | 0.099 |
| TCF7 | BCL2A1 | 0.074 |
| NFKB1 | BCL2A1 | 0.072 |
| RELB | BCL2A1 | 0.071 |
| TBX21 | BCL2A1 | 0.042 |
| RELA | BCL2A1 | 0.039 |
| EOMES | BCL2A1 | 0.036 |
| BCL6 | BCL2A1 | 0.024 |
| TOX | BCL2A1 | 0.007 |

**Table S5. Clonal expansion, clone size, and clonal persistence — GSE179994.** Patient-level paired Wilcoxon; Cohen's d_paired; values are patient-level medians; raw p.

| **Metric** | **Timepoint** | **BCL2A1high** | **BCL2A1low** | **Cohen_d** | **P_value** | **n** |
| --- | --- | --- | --- | --- | --- | --- |
| % cells in expanded clones (≥2) | Pre | 17.3 | 54.9 | −2.63 | 3.05E-05 | 16 |
| % cells in expanded clones (≥2) | Post | 28.1 | 68.1 | −2.16 | 7.81E-03 | 8 |
| % cells in expanded clones (≥3) | Pre | 0 | 42.9 | −1.94 | 6.10E-05 | 16 |
| % cells in expanded clones (≥3) | Post | 11.6 | 59.8 | −2.74 | 7.81E-03 | 8 |
| Mean clone size (cells/clone) | Pre | 1.11 | 1.78 | −0.70 | 3.05E-05 | 16 |
| Mean clone size (cells/clone) | Post | 1.22 | 2.39 | −0.62 | 7.81E-03 | 8 |
| Jaccard overlap (pre vs post) | — | 0.10 | 0.12 | −0.61 | 1.95E-01 | 8 |
| Fraction of pre-clones persisting | — | 0.30 | 0.35 | −0.30 | 3.12E-01 | 8 |

**Table S6. Single-cell metabolic flux (scFEA) in *BCL2A1*^high^ versus *BCL2A1*^low^ CD8⁺ tumor-infiltrating lymphocytes, discovery cohort (GSE176021).** Per-cell metabolic flux was estimated with the scFEA Human-M168 model and aggregated into 22 metabolic super-modules. For each super-module, flux was compared at the patient level (n = 11; two-sided paired Wilcoxon signed-rank test; Cohen's d_paired; Benjamini–Hochberg correction across the 22 super-modules). Rows are ordered by mean flux difference (high − low). Abbreviations: BCAA, branched-chain amino acid; GAG, glycosaminoglycan; HA, hyaluronic acid; PRPP, 5-phosphoribosyl-1-pyrophosphate; SM, super-module; TCA, tricarboxylic acid; TIL, tumor-infiltrating lymphocyte.

| **SM_id** | **SM_name** | **modules** | **n** | **High_mean** | **Low_mean** | **Diff** | **d_paired** | **p** | **p_adj** |
| --- | --- | --- | --- | --- | --- | --- | --- | --- | --- |
| SM03 | G3P → PRPP (glycerolipid/PRPP node) | 1 | 11 | 2.184E-03 | 3.679E-03 | -1.495E-03 | -0.911 | 2.44E-02 | 1.34E-01 |
| SM01 | Glycolysis (Glucose → Oxaloacetate) | 14 | 11 | 1.704E-02 | 1.779E-02 | -7.513E-04 | -0.495 | 4.20E-02 | 1.54E-01 |
| SM04 | TCA cycle (Acetyl-CoA → Acetyl-CoA) | 2 | 11 | 3.541E-03 | 4.121E-03 | -5.800E-04 | -1.011 | 1.86E-02 | 1.34E-01 |
| SM09 | BCAA (Leucine → Acetyl-CoA) | 8 | 11 | 4.853E-03 | 5.347E-03 | -4.946E-04 | -1.145 | 9.77E-04 | 2.15E-02 |
| SM20 | Purine metabolism (PRPP + Gln → GMP) | 18 | 11 | 4.637E-03 | 4.787E-03 | -1.498E-04 | -0.651 | 6.74E-02 | 2.12E-01 |
| SM07 | Propanoyl-CoA → Acetyl-CoA | 2 | 11 | 4.614E-03 | 4.691E-03 | -7.736E-05 | -0.420 | 3.20E-01 | 5.42E-01 |
| SM16 | N-glycan biosynthesis (Acetyl-CoA → glycan) | 12 | 11 | 5.297E-03 | 5.362E-03 | -6.548E-05 | -0.409 | 1.47E-01 | 2.95E-01 |
| SM21 | Pyrimidine metabolism (PRPP → dCMP) | 18 | 11 | 5.538E-03 | 5.598E-03 | -5.937E-05 | -0.229 | 5.77E-01 | 7.79E-01 |
| SM02 | Ser/Gly/Met metabolism (3PD → Propanoyl-CoA) | 18 | 11 | 5.795E-03 | 5.850E-03 | -5.502E-05 | -0.246 | 3.20E-01 | 5.42E-01 |
| SM12 | FA biosynthesis (Glucose → Fatty Acid) | 32 | 11 | 5.156E-03 | 5.200E-03 | -4.365E-05 | -0.477 | 1.47E-01 | 2.95E-01 |
| SM13 | Hyaluronic acid biosynthesis (Glucose → HA) | 5 | 11 | 4.115E-04 | 4.416E-04 | -3.015E-05 | -0.495 | 1.47E-01 | 2.95E-01 |
| SM18 | Dermatan sulfate (GAG) | 3 | 11 | 2.308E-02 | 2.311E-02 | -2.076E-05 | -0.212 | 5.20E-01 | 7.62E-01 |
| SM08 | Glutamate metabolism | 5 | 11 | 6.789E-07 | 8.696E-06 | -8.017E-06 | -0.605 | 4.20E-02 | 1.54E-01 |
| SM10 | Polyamine (Putrescine → Glyoxylate + pyruvate) | 8 | 11 | 2.317E-03 | 2.319E-03 | -2.098E-06 | -0.304 | 4.65E-01 | 7.30E-01 |
| SM17 | O-glycan biosynthesis (Dolichyl-P + Ser/Thr) | 4 | 11 | 1.592E-03 | 1.593E-03 | -2.795E-07 | -0.272 | 6.38E-01 | 7.79E-01 |
| SM06 | Aspartate → β-Alanine | 5 | 11 | 2.184E-03 | 2.184E-03 | -5.597E-08 | -0.155 | 7.00E-01 | 8.11E-01 |
| SM15 | Sialic acid (UDP-GlcNAc → CMP-NeuNAc) | 1 | 11 | 4.422E-05 | 4.424E-05 | -2.336E-08 | -0.733 | 2.44E-02 | 1.34E-01 |
| SM14 | Glycogen biosynthesis (G1P → Glycogen) | 1 | 11 | 4.245E-03 | 4.244E-03 | 6.332E-07 | 0.399 | 8.98E-01 | 8.98E-01 |
| SM19 | Heparan sulfate (GAG) | 1 | 11 | 5.642E-03 | 5.639E-03 | 3.491E-06 | 0.119 | 8.31E-01 | 8.71E-01 |
| SM05 | Aspartate → Oxaloacetate | 5 | 11 | 4.717E-03 | 4.714E-03 | 3.534E-06 | 0.556 | 1.02E-01 | 2.79E-01 |
| SM11 | Polyamine (Putrescine → Spermine) | 2 | 11 | 2.659E-05 | 1.405E-05 | 1.254E-05 | 0.336 | 6.38E-01 | 7.79E-01 |
| SM22 | Sterol biosynthesis (Farnesyl-PP → cholesterol) | 3 | 11 | 9.347E-03 | 8.954E-03 | 3.930E-04 | 0.255 | 8.31E-01 | 8.71E-01 |

**Table S7. Single-cell metabolic flux (scFEA) in *BCL2A1*^high^ versus *BCL2A1*^low^ CD8⁺ TILs across treatment, longitudinal cohort (GSE179994)**. Super-module flux (as in Table S6) was compared between groups separately before treatment (n = 18) and after treatment (n = 8), with the change in effect between timepoints and whether the direction of difference is preserved. The change in flux difference between timepoints orders rows. Abbreviations: BCAA, branched-chain amino acid; GAG, glycosaminoglycan; HA, hyaluronic acid; PRPP, 5-phosphoribosyl-1-pyrophosphate; SM, super-module; TCA, tricarboxylic acid; TIL, tumor-infiltrating lymphocyte.

| **SM_id** | **SM_name** | **Diff_PRE** | **p_adj_PRE** | **Diff_POST** | **p_adj_POST** | **Diff_change** | **consistent** |
| --- | --- | --- | --- | --- | --- | --- | --- |
| SM03 | G3P → PRPP (glycerolipid/PRPP node) | 3.196E-04 | 7.78E-01 | -3.802E-04 | 6.68E-01 | -6.998E-04 | FALSE |
| SM06 | Aspartate → β-Alanine | 9.460E-05 | 1.00E+00 | -1.017E-04 | 1.43E-01 | -1.963E-04 | FALSE |
| SM09 | BCAA (Leucine → Acetyl-CoA) | -8.918E-05 | 7.78E-01 | -2.212E-04 | 4.30E-02 | -1.321E-04 | TRUE |
| SM12 | FA biosynthesis (Glucose → Fatty Acid) | 6.148E-06 | 1.00E+00 | -4.102E-05 | 4.91E-01 | -4.717E-05 | FALSE |
| SM16 | N-glycan biosynthesis (Acetyl-CoA → glycan) | 2.249E-05 | 7.78E-01 | -5.579E-06 | 9.45E-01 | -2.807E-05 | FALSE |
| SM08 | Glutamate metabolism | -2.955E-06 | 7.89E-01 | -1.787E-05 | 4.30E-02 | -1.492E-05 | TRUE |
| SM18 | Dermatan sulfate (GAG) | -4.711E-07 | 7.78E-01 | -2.441E-06 | 4.91E-01 | -1.970E-06 | TRUE |
| SM07 | Propanoyl-CoA → Acetyl-CoA | -2.371E-07 | 4.35E-01 | -2.470E-07 | 4.91E-01 | -9.827E-09 | TRUE |
| SM19 | Heparan sulfate (GAG) | -7.985E-07 | 1.00E+00 | 1.092E-06 | 1.50E-01 | 1.891E-06 | FALSE |
| SM05 | Aspartate → Oxaloacetate | -8.209E-06 | 6.16E-02 | -4.330E-06 | 1.50E-01 | 3.880E-06 | TRUE |
| SM13 | Hyaluronic acid biosynthesis (Glucose → HA) | -5.509E-05 | 3.30E-01 | -4.906E-05 | 4.30E-02 | 6.027E-06 | TRUE |
| SM11 | Polyamine (Putrescine → Spermine) | -2.867E-06 | 1.00E+00 | 3.986E-06 | 8.84E-01 | 6.853E-06 | FALSE |
| SM14 | Glycogen biosynthesis (G1P → Glycogen) | -1.546E-05 | 1.00E+00 | 1.841E-08 | 4.91E-01 | 1.548E-05 | FALSE |
| SM21 | Pyrimidine metabolism (PRPP → dCMP) | -6.699E-05 | 5.82E-01 | -4.086E-05 | 5.61E-01 | 2.614E-05 | TRUE |
| SM10 | Polyamine (Putrescine → Glyoxylate + pyruvate) | -3.752E-06 | 7.89E-01 | 2.845E-05 | 1.43E-01 | 3.220E-05 | FALSE |
| SM15 | Sialic acid (UDP-GlcNAc → CMP-NeuNAc) | -2.613E-05 | 7.89E-01 | 2.276E-05 | 6.68E-01 | 4.889E-05 | FALSE |
| SM20 | Purine metabolism (PRPP + Gln → GMP) | -3.158E-04 | 1.17E-01 | -2.174E-04 | 4.30E-02 | 9.835E-05 | TRUE |
| SM17 | O-glycan biosynthesis (Dolichyl-P + Ser/Thr) | -7.676E-05 | 1.17E-03 | 3.524E-05 | 8.84E-01 | 1.120E-04 | FALSE |
| SM02 | Ser/Gly/Met metabolism (3PD → Propanoyl-CoA) | -9.835E-05 | 5.82E-01 | 5.828E-05 | 7.42E-01 | 1.566E-04 | FALSE |
| SM22 | Sterol biosynthesis (Farnesyl-PP → cholesterol) | -3.851E-04 | 5.82E-01 | -1.818E-04 | 6.34E-01 | 2.033E-04 | TRUE |
| SM01 | Glycolysis (Glucose → Oxaloacetate) | -5.742E-04 | 3.95E-01 | -3.177E-04 | 4.77E-01 | 2.565E-04 | TRUE |
| SM04 | TCA cycle (Acetyl-CoA → Acetyl-CoA) | -6.041E-05 | 5.82E-01 | 2.171E-04 | 4.91E-01 | 2.775E-04 | FALSE |

**Table S8. Single-cell metabolic flux (scFEA) in *BCL2A1*^high^ versus *BCL2A1*^low^ CD8⁺ TILs, independent validation cohort (GSE243013).** Super-module flux compared at the patient level (n = 57). The reduced branched-chain amino acid and purine flux in *BCL2A1*^high^ cells reproduces independently in this post-treatment cohort. Rows are ordered by mean flux difference (high − low).

Abbreviations: BCAA, branched-chain amino acid; GAG, glycosaminoglycan; HA, hyaluronic acid; PRPP, 5-phosphoribosyl-1-pyrophosphate; SM, super-module; TCA, tricarboxylic acid; TIL, tumor-infiltrating lymphocyte.

| **SM_id** | **SM_name** | **modules** | **High_mean** | **Low_mean** | **Diff** | **d_paired** | **p** | **p_adj** |
| --- | --- | --- | --- | --- | --- | --- | --- | --- |
| SM03 | G3P → PRPP (glycerolipid/PRPP node) | 1 | 3.379E-03 | 4.388E-03 | -1.009E-03 | -0.379 | 3.76E-05 | 2.76E-04 |
| SM01 | Glycolysis (Glucose → Oxaloacetate) | 14 | 1.482E-02 | 1.545E-02 | -6.328E-04 | -0.536 | 2.72E-05 | 2.76E-04 |
| SM09 | BCAA (Leucine → Acetyl-CoA) | 8 | 6.743E-03 | 7.055E-03 | -3.120E-04 | -0.509 | 3.39E-04 | 1.18E-03 |
| SM04 | TCA cycle (Acetyl-CoA → Acetyl-CoA) | 2 | 1.142E-03 | 1.422E-03 | -2.796E-04 | -0.262 | 2.72E-05 | 2.76E-04 |
| SM22 | Sterol biosynthesis (Farnesyl-PP → cholesterol) | 3 | 1.435E-02 | 1.456E-02 | -2.140E-04 | -0.223 | 8.92E-02 | 1.23E-01 |
| SM21 | Pyrimidine metabolism (PRPP → dCMP) | 18 | 7.056E-03 | 7.153E-03 | -9.680E-05 | -0.268 | 2.77E-04 | 1.18E-03 |
| SM07 | Propanoyl-CoA → Acetyl-CoA | 2 | 3.733E-03 | 3.827E-03 | -9.442E-05 | -0.445 | 3.75E-04 | 1.18E-03 |
| SM16 | N-glycan biosynthesis (Acetyl-CoA → glycan) | 12 | 7.945E-03 | 8.034E-03 | -8.906E-05 | -0.273 | 2.00E-02 | 4.17E-02 |
| SM20 | Purine metabolism (PRPP + Gln → GMP) | 18 | 4.588E-03 | 4.670E-03 | -8.262E-05 | -0.516 | 8.47E-05 | 4.66E-04 |
| SM06 | Aspartate → β-Alanine | 5 | 5.470E-03 | 5.532E-03 | -6.138E-05 | -0.216 | 2.04E-02 | 4.17E-02 |
| SM11 | Polyamine (Putrescine → Spermine) | 2 | 4.329E-04 | 4.695E-04 | -3.660E-05 | -0.068 | 2.05E-01 | 2.51E-01 |
| SM08 | Glutamate metabolism | 5 | 1.085E-04 | 1.279E-04 | -1.939E-05 | -0.221 | 2.09E-02 | 4.17E-02 |
| SM02 | Ser/Gly/Met metabolism (3PD → Propanoyl-CoA) | 18 | 5.480E-03 | 5.490E-03 | -1.033E-05 | -0.175 | 5.41E-02 | 8.49E-02 |
| SM15 | Sialic acid (UDP-GlcNAc → CMP-NeuNAc) | 1 | 8.255E-04 | 8.320E-04 | -6.479E-06 | -0.068 | 9.54E-02 | 1.23E-01 |
| SM10 | Polyamine (Putrescine → Glyoxylate + pyruvate) | 8 | 2.157E-03 | 2.162E-03 | -5.307E-06 | -0.250 | 4.92E-02 | 8.48E-02 |
| SM12 | FA biosynthesis (Glucose → Fatty Acid) | 32 | 4.155E-03 | 4.159E-03 | -4.044E-06 | -0.105 | 5.01E-02 | 8.48E-02 |
| SM05 | Aspartate → Oxaloacetate | 5 | 7.034E-04 | 7.067E-04 | -3.307E-06 | -0.046 | 6.50E-02 | 9.53E-02 |
| SM14 | Glycogen biosynthesis (G1P → Glycogen) | 1 | 9.655E-03 | 9.655E-03 | -4.792E-07 | -0.090 | 5.49E-01 | 6.35E-01 |
| SM13 | Hyaluronic acid biosynthesis (Glucose → HA) | 5 | 5.486E-05 | 5.497E-05 | -1.100E-07 | -0.011 | 1.39E-02 | 3.83E-02 |
| SM19 | Heparan sulfate (GAG) | 1 | 2.135E-02 | 2.135E-02 | 3.504E-07 | 0.069 | 6.64E-01 | 7.31E-01 |
| SM18 | Dermatan sulfate (GAG) | 3 | 7.443E-03 | 7.425E-03 | 1.847E-05 | 0.082 | 8.13E-01 | 8.13E-01 |
| SM17 | O-glycan biosynthesis (Dolichyl-P + Ser/Thr) | 4 | 6.209E-03 | 6.140E-03 | 6.914E-05 | 0.142 | 7.52E-01 | 7.88E-01 |

**Table S9. Per-donor BCL2A1 flow cytometry measurements on gated CD8⁺ T cells.** BCL2A1⁺ percentage and median fluorescence intensity (MFI) on CD8⁺ T cells from three independent healthy donors under Resting, Activated, and TNF-α (5 ng mL⁻¹) conditions. All values are Attune NxT outputs on compensated FCS data.

| **Group** | **Donor** | **%BCL2A1⁺ in CD8⁺** | **MFI BCL2A1 on CD8⁺** |
| --- | --- | --- | --- |
| Resting | 1 | 2.3 | 3,400 |
| Resting | 2 | 4.8 | 3,988 |
| Resting | 3 | 1.4 | 2,988 |
| Activated | 1 | 4.1 | 4,151 |
| Activated | 2 | 1.0 | 3,199 |
| Activated | 3 | 5.2 | 3,850 |
| TNF-α 5 ng mL⁻¹ | 1 | 46.9 | 11,385 |
| TNF-α 5 ng mL⁻¹ | 2 | 43.9 | 10,950 |
| TNF-α 5 ng mL⁻¹ | 3 | 42.8 | 10,426 |

**Table S10. Group summary statistics for BCL2A1 flow cytometry validation**. Mean ± SD across three donors per group, with Tukey HSD pairwise comparisons versus Activated. One-way ANOVA: %BCL2A1⁺ F(2,6) = 416.41, P = 3.7 × 10⁻⁷; MFI F(2,6) = 223.78, P = 2.3 × 10⁻⁶.

| **Group** | **n** | **%BCL2A1⁺ (mean ± SD)** | ***P* %⁺ (vs Act.)** | **MFI (mean ± SD)** | **Fold MFI** |
| --- | --- | --- | --- | --- | --- |
| Resting | 3 | 2.83 ± 1.76% | 0.93 (ns) | 3,459 ± 503 | 0.93× |
| Activated | 3 | 3.43 ± 2.18% | — | 3,733 ± 487 | 1.00× |
| TNF-α 5 ng mL⁻¹ | 3 | 44.53 ± 2.12% | <0.0001 (***) | 10,920 ± 480 | 2.93× |

**Fig S1. Marker expression along the CD8⁺ T-cell pseudotime trajectory in GSE176021**. Log-normalized expression of *BCL2A1*, cytotoxic markers (*GZMB*, *PRF1*), and exhaustion-associated genes (*TOX*, *PDCD1*, *EOMES*) plotted across the pseudotime continuum, showing their distribution along early, intermediate, and late trajectory states.

**Fig S2. Paired dot plot of TCR repertoire metrics in *BCL2A1* high vs low CD8 T cells from GSE176021**. Each patient contributes one paired observation (connecting line). Bars denote patient-level medians. (A) Shannon entropy. (B) Richness per 100 cells. (C) % cells in expanded clones (size ≥2). (D) % cells in expanded clones (size ≥3). (E) Pielou evenness J. P-values from paired Wilcoxon signed-rank (two-sided exact); Cohen d_paired reported. Blue = Low, red = High.

**Fig S3. Regulon activity and correlation with *BCL2A1* expression in paired pre- and post-treatment samples (GSE179994). (A)** Heatmap of AUCell regulon activity for eight key TFs (*RELA*, *RELB*, *NFKB1*, *TCF7*, *TOX*, *TBX21*, *EOMES*, and *BCL6*) in *BCL2A1*^high^ versus *BCL2A1*^low^ CD8⁺ TILs, separated by treatment status. Scatter plots showing Spearman correlations between *BCL2A1* expression and regulon activity in *BCL2A1*^high^ cells pre-treatment **(B)** and post-treatment **(C)**.

A

C

B

high-pre	high-post	low-pre	low-post

**Figure S4. Gene expression profiles of *BCL2A1*^high^ versus *BCL2A1*^low^ CD8⁺ T cells in the validated cohort (GSE243013**)**.** Per-cell mean expression (log-normalized) in *BCL2A1*^high^ versus *BCL2A1*^low^ CD8⁺ T cells from the independent post-treatment cohort (GSE243013, n = 59). **(A)** Anti-apoptotic genes. (B) Pro-apoptotic genes. (C) Exhaustion and cytotoxic-effector genes. Color = mean expression (per-panel scale).

**Figure S5. CD8⁺ T cell subtype composition and per-subtype TCR repertoire by *BCL2A1* group (GSE243013).** CD8⁺ T cell subtypes in *BCL2A1*^high^ versus *BCL2A1*^low^ cells, independent post-treatment cohort (GSE243013, n = 59). Patient-level paired Wilcoxon with BH-FDR (n = 54 of 59 patients; 5 patients lacking *BCL2A1*^high^ or *BCL2A1*^low^ cells in the compared subtypes were excluded from the paired test). **(A)** Subtype proportion per group (stacked). Stars indicate patient-level enrichment or depletion in *BCL2A1*^high^ cells (paired Wilcoxon, BH-FDR). **(B)** Shannon entropy (bars) and percentage of cells in expanded clones (≥2, line) per subtype within the *BCL2A1*^high^ group, ordered by expansion; descriptive. * q<0.05, ** q<0.01, *** q<0.001; ns, not significant).

**Figure S6. Regulon importance and activity of transcription factors in *BCL2A1*^high^ versus *BCL2A1*^low^ CD8⁺ T cells in the validated cohort (GSE243013**)**.** Color codes represent *BCL2A1* expression groups: red for high and blue for low. **(A)** Bar plot showing regulon importance scores for eight transcription factors (*RELA*, *RELB*, *NFKB1*, *TCF7*, *TOX*, *TBX21*, *EOMES*, and *BCL6*) in *BCL2A1*^high^ (red) and *BCL2A1*^low^ (blue) CD8⁺ T cells. Importance scores were inferred using GRNBoost2. **(B)** Violin plots of AUCell scores showing the distribution of regulon activity for the same transcription factors across single cells in each group.

B

A

BB

**Figure S7. CD8⁺ T cell TCR repertoire diversity and clonal expansion by *BCL2A1* group (GSE243013).** TCR repertoire features in *BCL2A1*^high^ versus *BCL2A1*^low^ CD8⁺ T cells, independent post-treatment cohort (GSE243013, n = 59). **(A)** Shannon entropy. **(B)** Percentage of cells in expanded clones (size ≥2). **(C)** Percentage of cells in expanded clones (size ≥3). (D) Pielou evenness J′. Each point is one patient; boxes show median and interquartile range. Patient-level paired Wilcoxon, n = 56 paired patients; *** p<0.001.
